# Tuning selectivity of electrochemical reactions by atomically dispersed platinum catalyst

**DOI:** 10.1038/ncomms10922

**Published:** 2016-03-08

**Authors:** Chang Hyuck Choi, Minho Kim, Han Chang Kwon, Sung June Cho, Seongho Yun, Hee-Tak Kim, Karl J. J. Mayrhofer, Hyungjun Kim, Minkee Choi

**Affiliations:** 1 Department of Chemical and Biomolecular Engineering, Korea Advanced Institute of Science and Technology, Daejeon 305-701, Korea; 2 Department of Interface Chemistry and Surface Engineering, Max-Planck-Institut für Eisenforschung GmbH, Max-Planck-Strasse 1, 40237 Düsseldorf, Germany; 3 Graduate School of EEWS, Korea Advanced Institute of Science and Technology, Daejeon 305-701, Korea; 4 Department of Applied Chemical Engineering, Chonnam National University, Yongbong 300, Buk-gu, Gwangju 500-757, Korea; 5 Forschungszentrum Jülich, ‘Helmholtz-Institut Erlangen-Nürnberg' (IEK 11), Nägelsbachstrasse 49b, 91052 Erlangen, Germany

## Abstract

Maximum atom efficiency as well as distinct chemoselectivity is expected for electrocatalysis on atomically dispersed (or single site) metal centres, but its realization remains challenging so far, because carbon, as the most widely used electrocatalyst support, cannot effectively stabilize them. Here we report that a sulfur-doped zeolite-templated carbon, simultaneously exhibiting large sulfur content (17 wt% S), as well as a unique carbon structure (that is, highly curved three-dimensional networks of graphene nanoribbons), can stabilize a relatively high loading of platinum (5 wt%) in the form of highly dispersed species including site isolated atoms. In the oxygen reduction reaction, this catalyst does not follow a conventional four-electron pathway producing H_2_O, but selectively produces H_2_O_2_ even over extended times without significant degradation of the activity. Thus, this approach constitutes a potentially promising route for producing important fine chemical H_2_O_2_, and also offers opportunities for tuning the selectivity of other electrochemical reactions on various metal catalysts.

## Abstract (web summary)

Atomically dispersed metal catalysts display high atom efficiency for electrocatalytic processes. Here, the authors report that sulfur-doped zeolite-templated carbon stabilizes highly dispersed platinum species, predominantly as single-atom centres, and probe its oxygen reduction selectivity.

## Graphical abstract

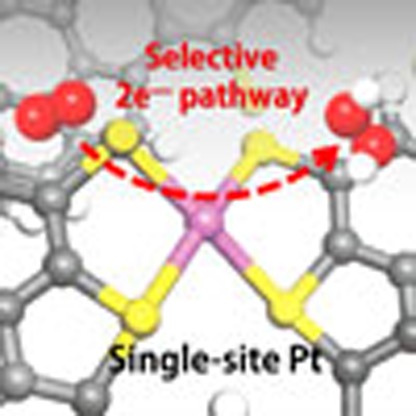

Noble metals are the most widely used catalysts in industry because of their high activity, selectivity and stability in many important reactions. The catalysts often consist of finely dispersed metal nanoclusters on porous supports to maximize the active surface area. In principle, as the size of the metal cluster further decreases to a sub-nanometre scale and ultimately to an atomic level (atomically dispersed metal catalyst), the catalytic properties are drastically changed^1^,^2^. Increased interaction with a support can modulate the electronic properties of metal catalysts and reduced metal–metal coordination can suppress chemical reactions requiring multi-atom sites. This can provide unique opportunities for tuning activity and chemoselectivity^3^. Because the atomically dispersed metal catalysts (they are also called ‘single-atom catalysts' in the literature) are highly unstable and prone to agglomeration due to their high surface energy, metal oxide supports (or alloys^4^,^5^,^6^,^7^) providing strong metal–support interactions, for example, CeO_2_ (refs 8, 9, 10, 11, 12, 13), FeO_*x*_ (refs 14, 15, 16, 17, 18), TiO_2_ (refs 19, 20), Al_2_O_3_ (refs 21, 22, 23, 24, 25) and silica/zeolite with and without alkali ions^26^,^27^,^28^,^29^,^30^,^31^,^32^,^33^, are typically indispensable for their stabilization.

In principle, atomically dispersed metal catalysts can exhibit distinct properties not only in chemical reactions but also in electrochemical reactions. Unfortunately, metal oxide supports are generally insulators or semiconductors with low electron conductivities. Moreover, they are unstable under corrosive electrochemical operating conditions^34^. In contrast, carbon-based materials can provide outstanding stability, good electron conductivity and large surface area, and are therefore considered as ideal candidates for supports in electrocatalysis. The carbons, however, show inert characteristics and do not provide strong metal–support interactions^35^. Hence, surface modifications (for example, oxidation) are often required to stabilize metal clusters even of a few nanometres in diameter^36^. Recently, highly sophisticated techniques such as atomic layer deposition^37^,^38^, mass-selected soft landing^39^ and arc discharge^40^ have been used to prepare sub-nanometre noble metal clusters on carbon surfaces. Nevertheless, these techniques still generate physical mixtures of atomically dispersed species as well as nanoscale clusters, which render the interpretation of the electrocatalytic performance rather vague.

In the present work, atomically dispersed Pt catalyst is selectively synthesized via a simple wet-impregnation method on zeolite-templated carbon (ZTC) containing an extra-large amount (up to 17 wt%) of sulfur. The abundant S-functionalities and unique carbon structure (that is, highly curved three-dimensional networks of graphene nanoribbons) can stabilize a relatively high loading of Pt (5 wt%) in the form of atomically dispersed Pt. It is notable that, in earlier works^11^,^12^,^13^,^14^,^15^,^16^,^17^,^19^,^20^,^21^,^22^,^23^,^24^,^27^,^28^,^29^,^30^,^31^,^41^, atomically dispersed Pt (or noble metals) could be predominantly synthesized only at very low loadings (<1 wt%) due to the small surface area of support materials as well as the lack of strong interaction between Pt and supports. In the oxygen reduction reaction (ORR) under an acidic environment, the catalyst does not follow the typical four-electron pathway producing H_2_O, but predominantly produce H_2_O_2_ via a two-electron pathway without promoting consecutive H_2_O_2_ decomposition reactions. Consequently, the highly dispersed Pt catalyst including site isolated atoms holds promise for the production of the important fine chemical H_2_O_2_ by electrochemical means in fuel cells.

## Results

### Preparation of ZTCs

S-doped ZTCs were synthesized by chemical vapour deposition (CVD) of acetylene/H_2_S in NaX zeolite at 823 K (see details in the Methods section). Subsequently, the samples were heat-treated at 1,073 K under two different gas atmospheres, that is, H_2_S/He and pure He. The former produced a carbon with a high S-content (HSC) and the latter produced a carbon with a low S-content (LSC). Dissolution of the zeolite template using an HCl/HF solution produced self-standing microporous carbon materials. Purely carbonaceous ZTC was also synthesized by acetylene CVD without any H_2_S flow for comparison^42^. N_2_ adsorption–desorption isotherms and structural properties of the carbon materials are provided in Supplementary Fig. 1 and Supplementary Table 1. Briefly, all the carbon materials show large surface areas of 2,400–2,800 m^2^ g^−1^ and micropore volumes of 0.95–1.04 ml g^−1^, indicating a faithful replication of the zeolite as a carbon framework^42^,^43^,^44^.

**Figure 1:**
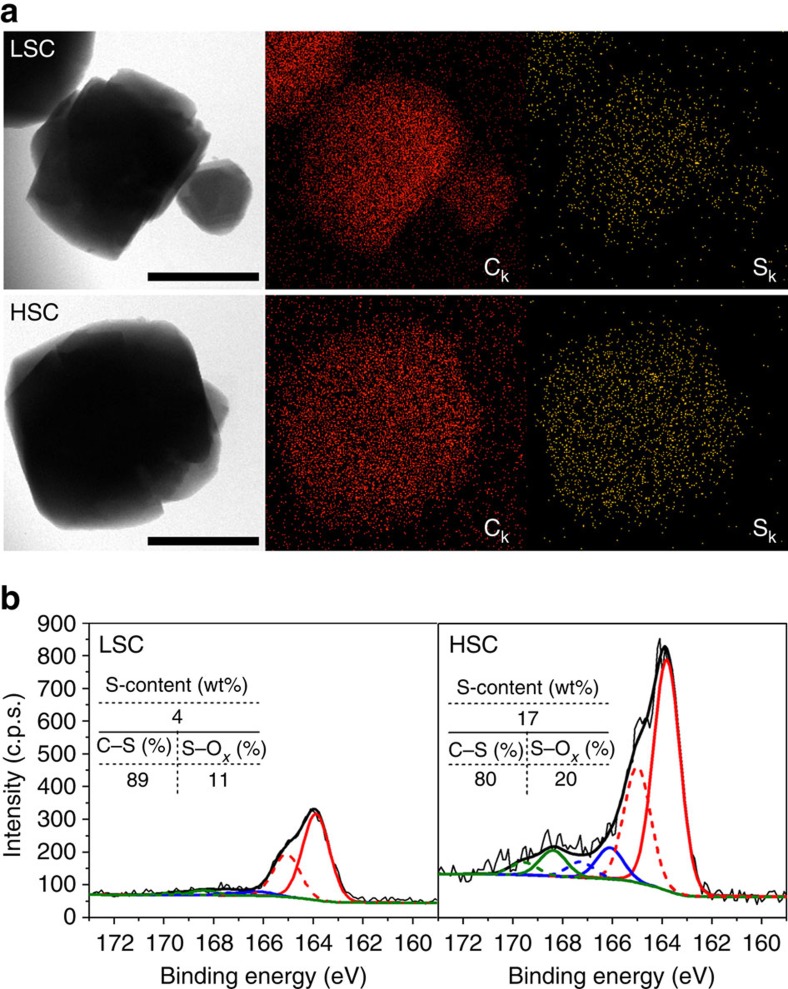
Characterizations of LSC and HSC. (**a**) Energy dispersive X-ray mapping images of C (red) and S (yellow); scale bar, 1.0 μm. (**b**) XPS-S_2*p*_ spectra. XPS peaks were deconvoluted with C–S (red lines) and S–O_*x*_ (blue and green lines) phases by considering a spin–orbit splitting of 1.2 eV in 2*p*_1/2_ (dotted lines) and 2*p*_3/2_ (solid lines). S-contents estimated from an elemental analysis and portions of S-phases determined from XPS peak deconvolution are summarized as an inset table.

**Figure 2:**
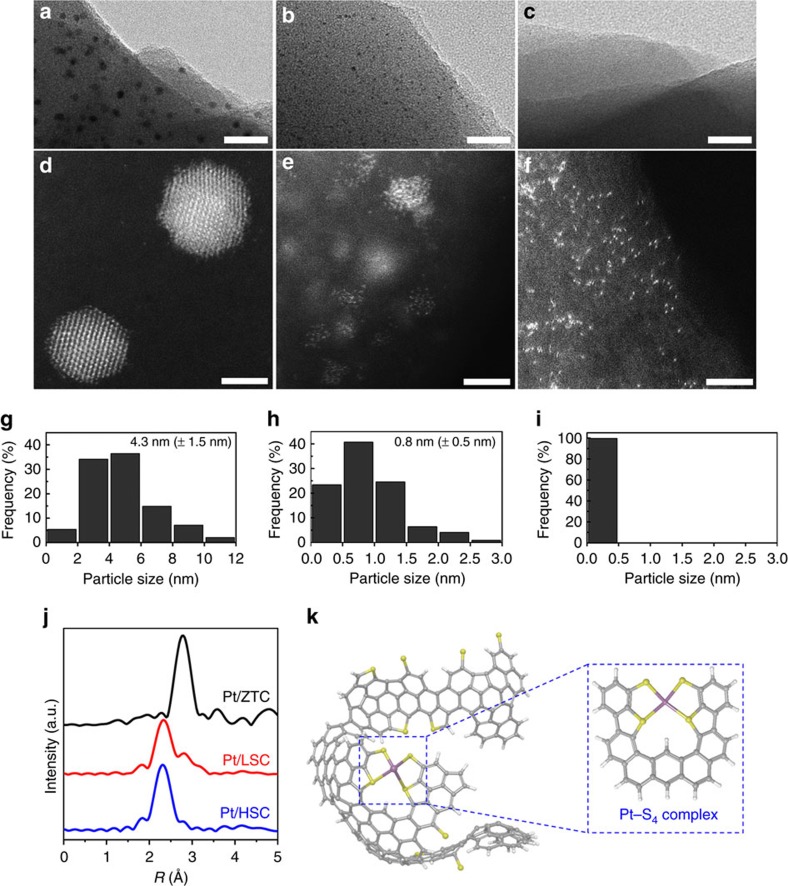
Structures of the Pt species on the prepared catalysts. (**a**–**c**) TEM images indicating the presence of ca. 4-nm Pt clusters in Pt/ZTC (**a**), Pt clusters (1–2 nm) in Pt/LSC (**b**), and the absence of discernable Pt species in Pt/HSC (**c**); scale bar, 30 nm. (**d**–**f**) To visualize the dispersion and structure of sub-nanometre Pt species, atomic resolution HAADF-STEM images are additionally provided for Pt/ZTC (**d**), Pt/LSC (**e**), and Pt/HSC (**f**); scale bar, 2 nm. (**g**–**i**) Histograms of the particle size distributions for Pt/ZTC (**g**), Pt/LSC (**h**), and Pt/HSC (**i**). Average particle sizes and s.d.'s of Pt/ZTC and Pt/LSC were fitted with a Gaussian function. The images reveal that Pt/LSC contains both of atomically dispersed Pt species and Pt clusters. Pt/HSC contains mainly the atomically dispersed Pt species. (**j**) Fourier transforms of *k*^3^-weighted Pt L_III_-edge EXAFS, confirming that Pt species in Pt/HSC exhibit only Pt–S coordination (CN=3.8) without significant Pt–Pt coordination. (**k**) Proposed atomistic structure of the Pt/HSC based on the buckybowl-like structure of zeolite-templated carbon suggested by Nishihara *et al.*^48^ with possible thiophene- and thiolate-like functional groups at graphene edge sites, as well as coordinated Pt species (C: grey, H: white, S: yellow and Pt: purple).

S-contents in HSC and LSC were determined by elemental analysis to be 17 and 4 wt%, respectively. Energy dispersive X-ray spectroscopy indicated that S atoms are uniformly distributed in the HSC and LSC particles (Fig. 1a). X-ray photoelectron spectroscopy (XPS) was used to study the chemical nature of the S-moieties (Fig. 1b). The results showed that the most dominant peaks are due to C–S bonds (163.8 eV for 2*p*_3/2_), while there are also some minor peaks corresponding to S–O_*x*_ bonds (166.1–168.4 eV for 2*p*_3/2_). Physically deposited elemental sulfurs may also show peaks in a similar binding energy range with C–S bonds, but their presence could be excluded. Namely, CS_2_ extraction did not decrease the S-contents of both samples^45^. The determination of the most dominant S species among the various moieties containing C–S bonds solely based on XPS is ambiguous (for example, sulfides, mercaptanes and thiophenes). However, considering that the final heat-treatment temperature for the carbon synthesis was sufficiently high (1,073 K) for aliphatic sulfides and thiols to thermally decompose^46^, aromatic thiols and thiophenes are likely the prevailing functional groups on the carbon surface. To the best of our knowledge, the HSC sample is a carbon material simultaneously exhibiting the largest framework-sulfur content as well as surface area among various S-doped carbon materials reported to date^47^. This may be attributed to the unique structural features of ZTCs. It was previously proposed that the spatial constraint of zeolite micropores leads to the formation of buckybowl-like nanographenes linked into three-dimensional networks^48^, which provides a large number of graphene edge sites that can allow the incorporation of S-functional groups (Fig. 2k).

### Pt-supported ZTCs

Pt catalysts were synthesized by a conventional wet-impregnation of H_2_PtCl_6_ on the prepared carbon supports, followed by H_2_ reduction at 523 K. Pt loadings of all the catalysts were found to be 5.0±0.2 wt%. Bright-field transmission electron microscopy (TEM) images (Fig. 2a–c) show that Pt clusters having ca. 4-nm diameters are dispersed in Pt/ZTC (Fig. 2a), while 1–2-nm Pt clusters are present in Pt/LSC (Fig. 2b). In Pt/HSC (Fig. 2c), no Pt cluster is discernable in TEM. These results indicate that Pt dispersion increases as the S-content in the carbon framework increases. To further visualize sub-nanometre Pt species, atomic resolution high-angle annular dark-field scanning transmission electron microscopy (HAADF-STEM) was carried out (Fig. 2d–f) along with particle size distribution analysis (Fig. 2g–i). Consistent with the TEM study, Pt/ZTC contains Pt clusters of a few nanometres in diameter (Fig. 2d). In Pt/LSC (Fig. 2e), atomically dispersed Pt species co-exist along with small Pt clusters (<3 nm). In Pt/HSC (Fig. 2f), Pt exists mainly as an atomically dispersed species without appreciable Pt clustering (more images are provided in Supplementary Fig. 2). The Pt/HSC showed imperceptible H_2_ chemisorption at 323 K, supporting the negligible presence of Pt clusters on which H_2_ is dissociated and chemisorbed. We confirmed that 5 wt% Pt is the maximum Pt loading allowing the selective synthesis of atomically dispersed Pt species even in HSC, and further increase of Pt loading above 5 wt% can also lead to the co-formation of Pt clusters. The atomically dispersed species are quite stable under the irradiation of highly energetic electron beam (300 kV) and thus their dynamic motion could be recorded (Supplementary Movie 1). Thermal hopping of Pt to nearby sites was observed, but Pt sintering was not observed within the time scale of our investigation (∼5 min). The Pt species were also stable under ambient conditions for over a year (Supplementary Fig. 3), indicating that the S-moieties on the carbon support efficiently stabilize them.

Extended X-ray absorption fine structure (EXAFS) was used to analyse the chemical nature of the Pt species (Fig. 2j). EXAFS fitting of the Pt/ZTC shows a dominant peak at 2.75 Å corresponding to the Pt–Pt coordination with a coordination number (CN) of 9.2 (Supplementary Table 2). In clear contrast, Pt/HSC shows a dominant peak at 2.29 Å, which can be assigned to Pt–S coordination (CN=3.8). No appreciable Pt–Pt coordination was detected. These results support that Pt is dominantly present as an atomically dispersed Pt ligated by approximately four S-moieties. Pt/LSC shows intermediate behaviour with both Pt–Pt (CN=3.3) and Pt–S coordinations (CN=2.4). XPS-Pt_4*f*_ (Supplementary Fig. 4) analysis revealed that the fraction of Pt^2+^ becomes more dominant as the S-content of the carbon support increases: Pt/ZTC, Pt/LSC and Pt/HSC show percentage ratios of 73.3/12.3/14.4, 40.3/49.1/10.6 and 0/100/0 for Pt^0^/Pt^2+^/Pt^4+^, respectively. Thus, it can be concluded that the atomically dispersed Pt species have an oxidation state of Pt^2+^, while Pt/ZTC and Pt/LSC have significant portions of Pt^0^. X-ray absorption near-edge structure (XANES) (Supplementary Figs 5–7; Supplementary Table 3) also showed that the white line intensity and position (*E*_0_) gradually increased in the order of ZTC<LSC<HSC, confirming that more oxidized Pt species present on support with higher S-content. The white line intensity of Pt/HSC exactly corresponds to that of Pt^2+^ (Supplementary Fig. 8)^49^. In earlier works in the literature^9^,^14^,^16^,^22^,^29^, the oxidation state of atomically dispersed Pt was found to vary between +2 and +4 depending on the type of support.

In the Pt/HSC, it is remarkable that the S-moieties fixed in the solid carbon framework can form a four-coordinated mononuclear Pt complex similar to the homogeneous organometallic Pt complexes ligated with molecular ligands^50^. It appears that the unique structure of the ZTC, that is, curved three-dimensional networks of graphene nanoribbons, allows the formation of highly S-coordinated Pt structures (Fig. 2k). It was previously reported that the ZTCs are also physically flexible due to their unique structure^51^, which may allow local geometrical optimization of the carbon framework for effective Pt coordination. To confirm the importance of the carbon microstructure on the S-content and the formation of atomically dispersed Pt, we synthesized a mesoporous S-doped carbon using mesoporous SBA-15 as a template, and similarly supported 5 wt% Pt. The material showed only 7 wt% S-content and the dominant presence of Pt clusters (Supplementary Fig. 9). This indicates that the unique carbon structure of ZTC plays a decisive role for efficient doping of S and consequent stabilization of atomically dispersed Pt.

### Oxygen reduction reaction

**Figure 3:**
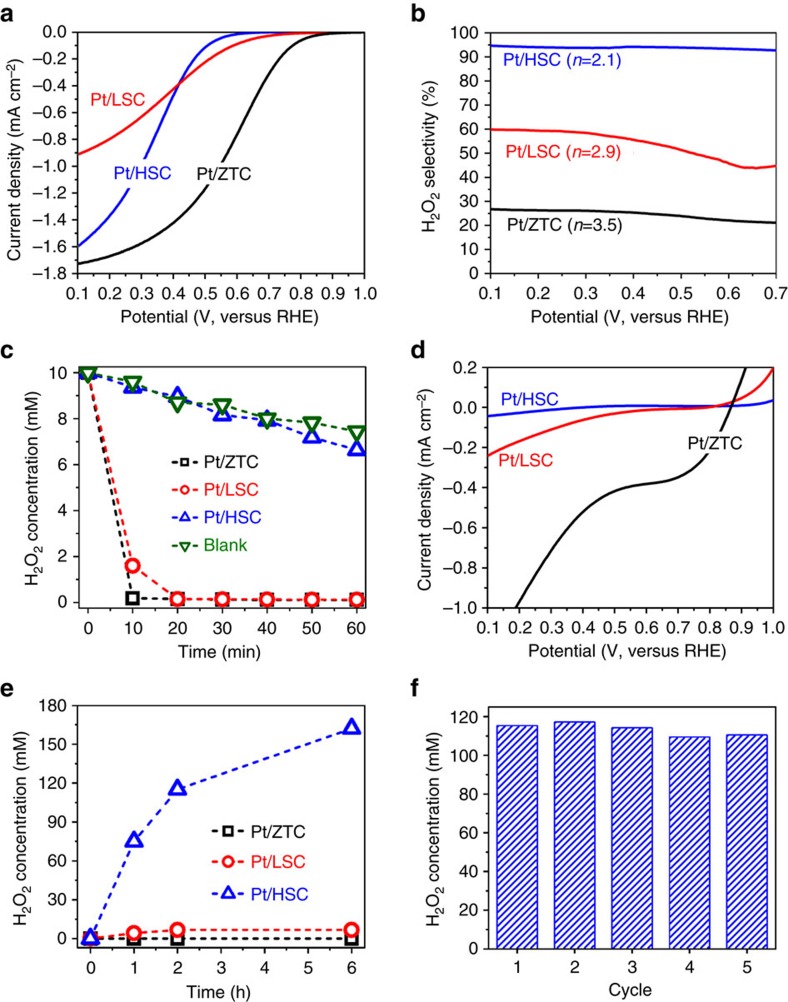
Electrocatalytic properties. (**a**) ORR activities of the prepared catalysts measured in an O_2_-saturated 0.1 M HClO_4_ electrolyte with 900 r.p.m. rotation. (**b**) H_2_O_2_ production selectivity estimated by RRDE experiments (Pt ring potential: 1.2 *V*_RHE_). (**c**) PDR activities derived from the decline in H_2_O_2_ concentration in a 10 mM H_2_O_2_ starting solution (50 ml) containing 10 mg catalysts. For comparison, the PDR of a blank solution without catalyst was also investigated. (**d**) PRR activities measured in a 10 mM H_2_O_2_/0.1 M HClO_4_ electrolyte with 900 r.p.m. rotation. (**e**) Accumulated H_2_O_2_ concentrations in an H-cell with a 1 M HClO_4_ electrolyte and a Nafion 115 membrane. The H-cell was operated in short-circuit condition (*V*=0) at 278 K. (**f**) Concentrations of H_2_O_2_ produced on the Pt/HSC in an electrochemical H-cell during repeated 2 h operation cycles. Concentrations of H_2_O_2_ are evaluated by iodometry.

The ORR behaviour was investigated in an O_2_-saturated 0.1 M HClO_4_ electrolyte (Fig. 3a; see details in Supplementary Figs 10 and 11 and Supplementary Note 1). Pt/ZTC and Pt/LSC show onset potentials (potential at −1 μA cm^−2^) of 0.99 and 0.95 *V*_RHE_, respectively, which are comparable to those of typical Pt electrocatalysts that mainly produce H_2_O via a four-electron pathway (*E*°_O2/H2O_=1.23 *V*_SHE_)^52^,^53^. In contrast, Pt/HSC reveals an onset potential of 0.71 *V*_RHE_, close to the thermodynamic potential of H_2_O_2_ production (*E*°_O2/H2O2_=0.69 *V*_SHE_). Rotating ring disk electrode (RRDE) experiments (Fig. 3b) revealed that Pt/HSC produces H_2_O_2_ with up to 96% selectivity, while Pt/ZTC and Pt/LSC exhibit H_2_O_2_ production selectivities lower than 28 and 60%, respectively. This indicates that Pt/HSC catalyses the ORR reaction predominately through a two-electron pathway (*n*=2.1), whereas Pt/ZTC (*n*=3.5) and Pt/LSC (*n*=2.9) enable mixed two- and four-electron pathways. The results are consistent with the previous report by Anderson's group^54^, where size-selected Pt_*n*_ clusters deposited on indium tin oxide were used as an ORR catalyst. The materials showed increased H_2_O_2_ selectivity as the Pt_*n*_ cluster size decreased, and a maximized H_2_O_2_ selectivity was observed with the smallest Pt_1_ species. Very recently, Lee and colleagues also reported that supporting 0.35 wt% Pt on TiN led to the formation of atomically dispersed Pt as well as a small amount of Pt clusters^41^, which showed H_2_O_2_ selectivity of 65%. They also mentioned that H_2_O_2_ selectivity can reach 90%, if the Pt loading decreases to 0.1 wt% where the Pt cluster formation is further suppressed.

The kinetics of consecutive H_2_O_2_ decomposition via a peroxide disproportionation reaction (PDR, H_2_O_2(aq)_→H_2_O_(l)_+0.5O_2(g)_) and peroxide reduction reaction (PRR, H_2_O_2(aq)_+2H^+^_(aq)_+2e^−^→2H_2_O_(l)_) on Pt is expected to be so fast that no H_2_O_2_ escapes from its surface^55^, which in turn suggests that the high yield of H_2_O_2_ could indeed be due to the unique characteristics of the Pt/HSC. However, in the presence of site-blocking spectator species and low Pt-content, H_2_O_2_ diffusion into the bulk of the electrolyte could be artificially favoured compared with its subsequent decomposition in the RRDE experiments^56^. To rule out an artificial origin of the high H_2_O_2_ yield on Pt/HSC, the suppressions of PDR and PRR are separately investigated with 10 mM H_2_O_2_ solutions. While Pt/ZTC and Pt/LSC induce a fast decrease in H_2_O_2_ concentrations when 10 mg samples are dispersed in the solution, the PDR activity (Fig. 3c) is inhibited on Pt/HSC. Furthermore, Pt/HSC shows significantly larger overpotential and lower reduction currents for the PRR than the other samples (Fig. 3d), confirming that the unique ORR behaviour of the Pt/HSC is not an artefact. To elucidate the catalytic active site of Pt/HSC, ORR on Pt-free HSC and on Pt/HSC under CN^−^ poisoning is additionally studied (Supplementary Fig. 12). Pt-free HSC exhibits a much larger overpotential than that of Pt/HSC, and its ORR performance is not significantly affected by CN^−^ poisoning. In contrast, the ORR activity on Pt/HSC is significantly decreased after CN^−^ poisoning due to the blockage of the active Pt sites. These results support that ORR on Pt/HSC is indeed catalysed by the atomically dispersed Pt species.

To confirm the concept of selective H_2_O_2_ production in a fuel cell via single-site electrocatalysis, an electrochemical H-cell was employed as a first prototype reactor (Fig. 3e). Pt/HSC showed 97.5 μmol h^−1^ cm^−2^ H_2_O_2_ production rate during the initial 1 h under short-circuit operation (*V*=0), while Pt/ZTC and Pt/LSC exhibited production rates of ∼0 and 5.3 μmol h^−1^ cm^−2^, respectively. In total, the concentrations of H_2_O_2_ produced on the Pt/ZTC and Pt/LSC were <7 mM in a catholyte (5 ml) after 6 h operation, while the concentration reached up to 160 mM with Pt/HSC. Pt/HSC shows stable catalytic performance as confirmed by the repeated 2 h operation cycles (Fig. 3f). HAADF-STEM micrographs of the Pt/HSC after the operation indicate that most of the atomically dispersed Pt species are preserved after the operation (Supplementary Fig. 13).

### Active site characterization and theoretical calculations

To elucidate the active site structure in Pt/HSC, energy minimization for the three proposed Pt–S_4_ model structures was carried out via density functional theory (DFT) using Perdew–Burke–Ernzerhof functional^57^ coupled with semiempirical dispersion correction^58^ (Supplementary Fig. 14). The three models include (1) Pt ligated by two thiophenes and two thiolates, (2) Pt ligated by one thiophene, one thiol and two thiolates, and (3) Pt ligated by two thiols and two thiolates. Because the oxidation state of Pt was confirmed to be +2 by XPS and XANES, two anionic thiolate ligands (−S^−^) were expected to exist within Pt/HSC for charge-balancing. The three model structures were used for multi-shell fitting of the EXAFS (Supplementary Figs 15–17). The results showed that the first model structure gave the best fitting (R-factor=0.0053) over the other two model structures (R-factors=0.0122 and 0.0138). Thus, DFT calculations were carried out using the first model structure (Fig. 2k) to understand the ORR mechanism.

**Figure 4:**
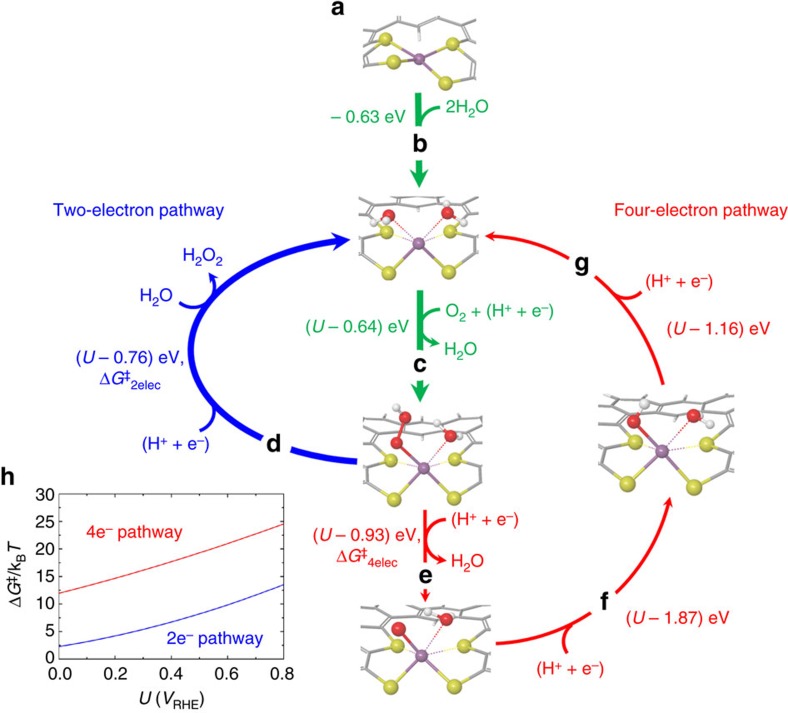
Proposed ORR mechanism on the atomically dispersed Pt catalyst. (**a**) Atomically dispersed Pt (purple) complexed with four sulfur-containing moieties consisting of two thiophene and two thiolate groups that are covalently bonded to a carbon framework. (**b**) Activation of Pt centre by substituting two S of thiophene-like moieties with two O of water molecules. (**c**) The first reduction of an O_2_ via proton-coupled electron transfer (PCET), forming OOH. This is a shared elementary step of the two- and four-electon pathways. (**d**) Two-electron pathway: H_2_O_2_ formation by the second PCET and the subsequent substitution of an H_2_O_2_ molecule with an outer-sphere H_2_O molecule, recovering the initial state where Pt is complexed with two thiolates and two waters. (**e**) Four-electron pathway: H_2_O formation by the second PCET involving O–O bond dissociation. (**f**) Four-electron pathway: OH formation by the third PCET to the O atom. (**g**) The fourth PCET to the OH forms an inner-sphere H_2_O, recovering the initial state where Pt is complexed with two thiolates and two waters. (**h**) Calculated kinetic barriers of the second PCET steps for the two-electron pathway (blue) and the four-electron pathway (red) using Marcus kinetic theory. (C: grey, H: white, S: yellow, O: red, and Pt: purple).

Since Pt^2+^ has a typical *d*^*8*^ electron configuration, the Pt–S_4_ complex forms a square-planar-type ligand arrangement (Fig. 4a). When it is solvated with water, it is found that the Pt^2+^ centre can favourably interact with two water molecules, while losing its interactions with two sulfur atoms of thiophene moieties (0.63 eV downhill; Fig. 4b). This is attributed to the stronger Lewis basicity of the oxygen lone pairs compared with that of the sulfur lone pairs in thiophene. This leads to a distorted Pt centre located out of the original square planar geometry, leading Pt to be more suitable for catalytic actions via ligand substitutions. By substituting one of the water molecules, the Pt centre then can reduce O_2_ via a series of proton-coupled electron transfers (PCETs)^59^. DFT energetics suggests that the first PCET (Fig. 4c) is the potential-determining step^60^ in both the two- and four-electron pathways, where the calculated ORR limiting potential is 0.64 *V*_RHE_, which relatively well matches with the experimental onset potential of 0.71 *V*_RHE_ considering the inherent error range of DFT energies and the simplicity of the model system.

This implies that all elementary steps of the four-electron pathway become thermodynamically favourable under the bias potential of *U*≤0.64 *V*_RHE_, and thereby the experimentally observed high chemoselectivity towards the two-electron pathway cannot be rationalized. Moreover, the competing step of the second PCET reaction towards the four-electron pathway (Fig. 4e) is thermodynamically even more favourable than that towards the two-electron pathway (Fig. 4d). The results indicate that electrocatalysis on atomically dispersed Pt catalyst is not controlled by thermodynamics, but rather is controlled by kinetics. We thus calculated the kinetic barriers required for the second PCETs using the Marcus theory. By taking the number of transferred proton–electron pair as a reaction coordinate for PCET, Marcus parabolas were constructed and the reorganization energies of *λ*_o_ were calculated (Supplementary Fig. 18). Then, the kinetic barrier of each pathway, Δ*G*^‡^ was calculated as a function of bias potential of *U*, using the Marcus theory:


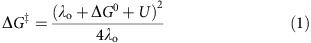


where Δ*G*^0^ is the Gibbs free energy of reaction with no bias potential. As shown in Fig. 4h, within considered potential range of *U*<0.8 *V*_RHE_, it is found that the two-electron pathway is always kinetically more favoured than the four-electron pathway. The main difference between the kinetics of the two- and four-electron pathways originates from the different *λ*_o_ values; *λ*_o_ of the two-electron pathway is only 0.55 eV, while *λ*_o_ of the four-electron pathway is 1.84 eV. The larger *λ*_o_ of the four-electron pathway is attributed to its second PCET accompanying a significant reorganization process for breaking the O–O bond.

Furthermore, the thermodynamic cost to bind H_2_O_2_ molecule to the Pt catalytic active centre is substantial; 0.41 eV uphill to bind one H_2_O_2_ molecule for PRR, and another 0.33 eV uphill to bind the second H_2_O_2_ molecule for PDR (Supplementary Fig. 19). This explains our experimental observation that the atomically dispersed Pt catalyst is almost inactive in decomposing H_2_O_2_.

## Discussion

We have synthesized a sulfur-doped ZTC (HSC), which exhibits extra-large S-content (17 wt% S) and surface area (∼2,800 m^2^ g^−1^). The novel carbon material stabilizes a relatively high loading of Pt (up to 5 wt%) in the form of atomically dispersed Pt. Its electrochemical behaviour significantly differs from that of cluster-type Pt catalysts: the atomically dispersed Pt catalyst selectively catalyse a two-electron ORR pathway producing H_2_O_2_, rather than the conventional four-electron ORR pathway producing H_2_O. Consequently, the catalyst can be considered as a promising approach for the production of H_2_O_2_, while at the same time generating electricity as a by-product of fuel cells. Moreover, the extraordinary electrochemical properties of the atomically dispersed Pt catalyst, demonstrated here for one sample reaction only, might be considered as a model for other important systems. As the abundant S-functionality in HSC allows the synthesis of various atomically dispersed metal catalysts other than Pt, it can provide wide material opportunities for studying unique catalytic behaviours in diverse electrochemical reactions.

## Methods

### Chemicals

All gases used in this study were supplied from Chungang Industrial Gases (Korea). H_2_PtCl_6_-5.5H_2_O was purchased from Kojima Chemicals (Japan). HClO_4_, KCN, CS_2_, starch solution (1% in H_2_O), Na_2_S_2_O_3_ and NaX zeolite were purchased from Sigma-Aldrich (USA). KI was supplied from Junsei Chemicals (Japan). H_2_O_2_ (30 wt%) and HCl (35 wt%) solutions were purchased from Daejung chemicals (Korea). HF (49 wt%) was supplied from J.T. Baker (USA).

### Preparation of catalysts

S-doped ZTCs were synthesized by CVD of acetylene/H_2_S mixed gas on NaX zeolite at 823 K. In a typical synthesis, 5 g NaX zeolite was loaded in a quartz plug-flow reactor. The reactor was heated to 823 K (2 K min^−1^ ramp) under 200 ml min^−1^ He flow. At the target temperature, 280 ml min^−1^ acetylene/H_2_S (1.4/1.4% in He) mixed gas was introduced into the reactor for 24 h. After CVD, the samples were heat-treated at 1,073 K (2 K min^−1^ ramp) for 3 h under two different gas atmospheres: 80 ml min^−1^ 5% H_2_S/He and pure He were flowed to produce carbons with HSC and LSC, respectively. For comparison, purely carbonaceous ZTC was similarly synthesized by acetylene CVD, but without H_2_S flow. After cooling to room temperature, the resulting samples were etched with 800 ml of an HCl/HF (1.1/0.8 wt%) aqueous solution twice to remove the zeolite template. After filtration and thorough washing with deionized water, the samples were dried at 373 K. Pt was supported on the prepared carbons by a conventional wet-impregnation method, followed by H_2_ reduction. Typically, 0.3 g carbon was dispersed in 100 ml deionized water containing 0.04 g of H_2_PtCl_6_-5.5H_2_O (5 wt% Pt loading). The water was evaporated at 353 K under 300 mbar. The resultant powder was further dried overnight in an oven at 353 K, and subsequently reduced at 523 K for 3 h under a 200 ml min^−1^ H_2_ flow.

### Physical characterizations

N_2_ adsorption–desorption isotherms were measured using a BEL-sorp-max (BEL Japan) volumetric analyser at 77 K. Before each measurement, all samples were degassed at 473 K for 4 h, and the specific areas were determined in the *P*/*P*_0_ range of 0.05–0.15 using a Brunauer–Emmett–Teller equation. The elemental analysis was conducted with a FLASH 2,000 series (Thermo Scientific). The inductively coupled plasma optical emission spectroscopy (ICP-OES) was carried out using an ICP-OES 720 (Agilent) after sample pretreatment at 473 K in a concentrated HNO_3_/HCl mixture (7:3). TEM and energy dispersive X-ray images were taken using a JEM2100-F (Jeol Ltd) operated at 200 kV accelerating voltage. HAADF-STEM analysis was performed using a FEI Titan cubed G2 60–300 microscope operating at 300 kV (Kaist Analysis centre for Research Advancement, KARA). The samples were prepared by drop casting and drying onto a lacey carbon grid. HRSTEM Z-Contrast conditions were achieved using a probe semi-angle of 19.3 mrad and an inner collection angle of the detector of 40 mrad. The Pt particle size distributions were analysed using an ImageJ software by counting at least 500 Pt species, and the average particle sizes and s.d.'s were fitted using a Gaussian function. H_2_ chemisorption isotherms was measured with ASAP2020 (Micromeritics) adsorption volumetric analyser. H_2_ (99.999%) gas was used as received. Before adsorption measurement, all the samples were re-reduced at 523 K flowing H_2_ (50 ml min^−1^) for 2 h, followed by evacuation for 2 h at the same temperature. Adsorption measurements were carried out at 323 K in the pressure range of 0.3–60 kPa. The chemisorption amounts were determined by extrapolation of the high-pressure linear portion (7–28 kPa) of the isotherm to zero pressure. XPS was carried out using Sigma Probe (Thermo VG Scientific) equipped with a microfocused monochromator X-ray source. XPS-S_2*p*_ and -Pt_4*f*_ peaks were deconvoluted with 1:2 and 3:4 of spin–orbit splitting ratios (Δ=1.2 eV for S_2*p*_ and 3.33 eV for Pt_4*f*_), respectively^61^. The binding energies used for the peak deconvolution of XPS-S spectra (for 2*p*_3/2_) were 163.8 eV for the C–S and 166.1/168.4 eV for the S–O_*x*_ species, respectively. The binding energies used for the peak deconvolution of the XPS-Pt spectra (for 4*f*_7/2_) were 71.2 eV for Pt^0^, 72.2 eV for Pt^2+^ and 73.5 eV for Pt^4+^, respectively.

### XAFS investigations

X-ray absorption fine spectroscopy (XAFS) data of the prepared catalysts over Pt L_III_-edge (11,570 eV) were collected in transmission mode using ionization detectors (Oxford) at the Pohang Accelerator Laboratory (7D-XAFS beamline). The beam energy and ring current were 2.5 GeV and 200 mA, respectively. The step and duration time for XANES and EXAFS were 0.3 eV and 2 s, and 0.03 Å^−1^ and 3 s, respectively. For XAFS measurement, 0.1 g catalysts were mounted in a sample holder (*w* × *l* × *d*=3 × 8 × 2 mm^3^). The XAFS data were calibrated using a Pt foil before and after the measurement to guarantee no shift in edge energy. The XANES data were analysed using Athena implemented in Demeter program package (0.9.23). In the fitting, the height for the arctangent function corresponding to the transition to continuum level was fixed to one to maintain the consistency in the analysis. For EXAFS analysis, Artemis also implemented in Demeter program package (0.9.23) was utilized after the data processing using Athena. The background removal was performed to extract EXAFS signal using AUTOBK program for *R*_bkg_=1 Å. The extracted EXAFS data in *k* space was Fourier transformed with the Kaiser–Bessel window function, 1 Å^−1^ after *k*^3^-weighting. Phase shifts and amplitude functions of the reference were generated using *Feff* 6L. The number of independent points of the data for the curve fit, *N*_idp_, determined from Nyquist theorem, was always larger than the number of variables, providing a sufficient degree of freedom, *N*_var_. The scattering path from the possible model structure was obtained from the *Feff* calculation. Only scattering with large contribution was included in the multi-shell fitting. The many-body reduction factor (*S*_0_^2^) for Pt was determined as 0.82 from the curve fit of the EXAFS of Pt foil. The statistical quality of the curve fit with the proposed models can be determined from the *R*-factor and the *χ*^2^ function available in the refinement.

### Electrochemical measurements

Electrochemical properties were characterized using VMP3 (Bio-Logic) in a three-electrode beaker cell equipped with a Pt wire counter electrode (ALS Co, 002233) and an Ag/AgCl reference electrode (ALS Co, 012167). Unless it is specially mentioned, 0.1 M HClO_4_ solution was used as an electrolyte. Before the electrochemical experiments, the Ag/AgCl reference electrode was calibrated in 0.1 M HClO_4_ electrolyte and potentials were converted to reversible hydrogen electrode (RHE) scale (*V*_RHE_=*V*_Ag/AgCl_+0.287 V).

The ORR was measured using RRDE-3A (ALS Co) as a rotator and a ring disk electrode (A-011162, ALS Co) as a working electrode. The catalyst ink was prepared by dispersing 5 mg catalysts in Nafion solution (6.5 ml, Nafion/catalyst=10%). The working electrode was prepared by dropping the catalyst ink (8.2 μl) onto the glassy carbon (4-mm diameter) of the ring disk electrode and then drying at room temperature. Loading amount of the catalysts was 50 μg cm^−2^ (2.5 μg_Pt_ cm^−2^). The ORR responses were recorded with a 10 mV s^−1^ scan rate in an O_2_-saturated electrolyte (rotating speed: 900 r.p.m., scan range: 0–1.1 *V*_RHE_). ORR results were presented after subtraction of the currents measured in an N_2_-saturated 0.1 M HClO_4_ electrolyte to remove capacitances. H_2_O_2_ selectivity and number of electrons transferred (*n*) were calculated from RRDE tests (equations 2 and 3), which were carried out by applying 1.2 *V*_RHE_ on the ring disk of the working electrode at a 900 r.p.m. speed.


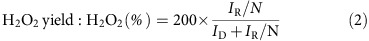



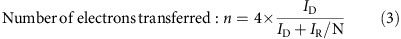


where *I*_R_ is the ring current, *I*_D_ is the disk current and *N* is the collection efficiency (0.2 of N was used after calibration). CO-stripping voltammetry was obtained from two cycles of cyclic voltammetry with a 50 mV s^−1^ scan rate (scan range: 0–1.2 *V*_RHE_). Before the CO-stripping analysis, CO molecules were adsorbed on Pt surface at 0.1 *V*_RHE_ for 30 min, and then N_2_ was purged into the electrolyte to remove dissolved CO gas.

The PDR was carried out by dispersing 10 mg catalysts in 10 mM H_2_O_2_ solution (50 ml). After specified time intervals, 5 ml solution was collected and then H_2_O_2_ concentration was determined by iodometry. The PRR was carried out following the same method for ORR except for the use of an N_2_-saturated 0.1 M HClO_4_ electrolyte containing 10 mM H_2_O_2_. CN^−^-poisoning test was also carried out following the same method for ORR but using an O_2_-saturated 0.1 M HClO_4_ electrolyte containing 10 mM KCN (precautions for safe handling of KCN in acid solution were considered).

H-cell was operated using 1 M HClO_4_ as both an anolyte and a catholyte (5 ml each), and the electrolytes were separated by a Nafion 115 membrane (3 × 5 cm^2^, DuPont). The working electrode (cathode) was prepared by spraying of the catalyst inks (50 mg catalysts+250 mg Nafion solution (5 wt%)+5 ml isopropyl alcohol) onto a carbon paper (W1S1005, CeTech). The catalyst loading was 2 mg cm^−2^ and the geometric active surface area of the electrode was 2 × 2 cm^2^. The commercially obtained Pt gas diffusion electrode (1 mg_Pt_ cm^−2^, Fuel Cell Earth) was used as an anode. The H-cell was operated at 278 K under short-circuit condition (*V*=0). H_2_ (100 ml min^−1^) and O_2_ (300 ml min^−1^) gases flowed at an ambient pressure to the anode and cathode channels, respectively. After reaction for specified time intervals, part of the catholyte was collected and then titrated by iodometry to determine H_2_O_2_ concentration.

### DFT calculations

Unrestricted DFT calculations were performed using Jaguar 8.2.12 (ref. 62) with the exchange–correlation function of Perdew–Burke–Ernzerhof combined with semiempirical dispersion correction of *ulg*. We used a LACVP** basis set describing Pt atom with LANL2DZ effective core basis set^63^, while the other atoms with a standard Pople's 6-31G** basis set.

We calculated Gibbs free energy change for each step using Born–Haber thermodynamic cycle scheme (Supplementary Fig. 20). The gas phase Gibbs free energy changes were calculated using Δ*G*^o^_gas_=Δ*E*_el._+*P*Δ*V*+(Δ*E*_trns._+Δ*E*_rot._+Δ*E*_vib._)−*T*(Δ*S*_trns._+Δ*S*_rot._+Δ*S*_vib._). Here Δ*E*_el._ is the electronic energy difference computed from DFT self-consistent field energies, Δ*E*_trns._ and Δ*S*_trns._ are translational energy and entropy difference, respectively, computed using ideal gas partition function, Δ*E*_rot._ and Δ*S*_rot._ are rotational energy and entropy difference, respectively, computed using rigid rotor partition function, and Δ*E*_vib._ and Δ*S*_vib._ are vibrational energy and entropy difference, respectively, computed using harmonic oscillator partition function coupled with vibrational frequency calculations (zero-point energy corrections are included). The solvation free energies, Δ*G*^o^_s_ were calculated using Poisson–Boltzmann implicit solvation method self-consistently coupled with DFT charge densities^64^,^65^. Here we set dielectric constant and probe radius to 80.37 and 1.40 Å, respectively, to account for the water solvation.

To assess the reaction free energy of redox reactions, the chemical potential of an electron, μ[e^−^] should be defined. To use the internal reference within the DFT calculation framework^66^,^67^, we calculated the absolute half-cell potential of ferrocene. This leads to *μ*[e^−^]=[−4.896−*U* (versus Fc/Fc^+^)] eV (ref. 68), which is converted into *μ*[e^−^]=[−4.516−*U* (versus RHE)] eV. The chemical potential of a proton, μ[H^+^], which is also required to fully assess the reaction free energy of PCET, is set as −11.152 eV that is comparable with the experimental proton solvation free energy of −10.95 to −11.92 eV (refs 69, 70). We note that these values yield 1.148 *V*_RHE_ for the DFT calculated thermodynamic potential of the four-electron pathway, and 0.700 *V*_RHE_ for the DFT calculated thermodynamic potential of the two-electron pathway in consistent with the experimental values.

## Additional information

**How to cite this article:** Choi, C. H. *et al.* Tuning selectivity of electrochemical reactions by atomically dispersed platinum catalyst. *Nat. Commun.* 7:10922 doi: 10.1038/ncomms10922 (2016).

## Supplementary Material

Supplementary Information Supplementary Figures 1-20, Supplementary Tables 1-3, Supplementary Note 1 and Supplementary References

Supplementary Movie 1 Dynamic motions of the atomically dispersed Pt species. The movie taken by HAADF-STEM at 300 kV showed thermal vibrations and hopping of the atomically dispersed Pt species to nearby sites. Notably, the Pt species were highly stable against metal sintering within the time scale of investigation.
