# Supplementary material for: Tuning selectivity of electrochemical reactions by atomically dispersed platinum catalyst
Source: Nat Commun. 2016 Mar 8;7:10922. doi: 10.1038/ncomms10922 (PMC4786782; doi:10.1038/ncomms10922)
Supplement: Supplementary Information — Supplementary Figures 1-20, Supplementary Tables 1-3, Supplementary Note 1 and Supplementary References [file ncomms10922-s1.pdf]

Supplementary Figures

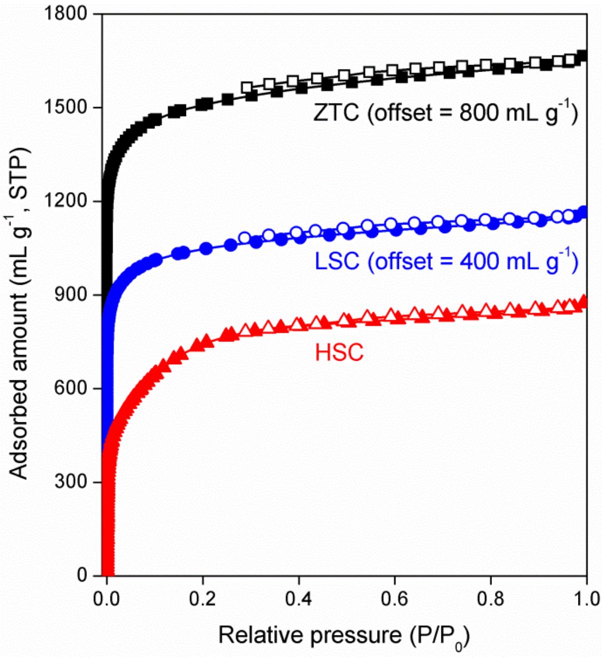

Supplementary Figure 1 | N_2 adsorption-desorption isotherms of the prepared carbon materials. Before measurements at 77 K, all the samples were degassed at 473 K for 4 h.

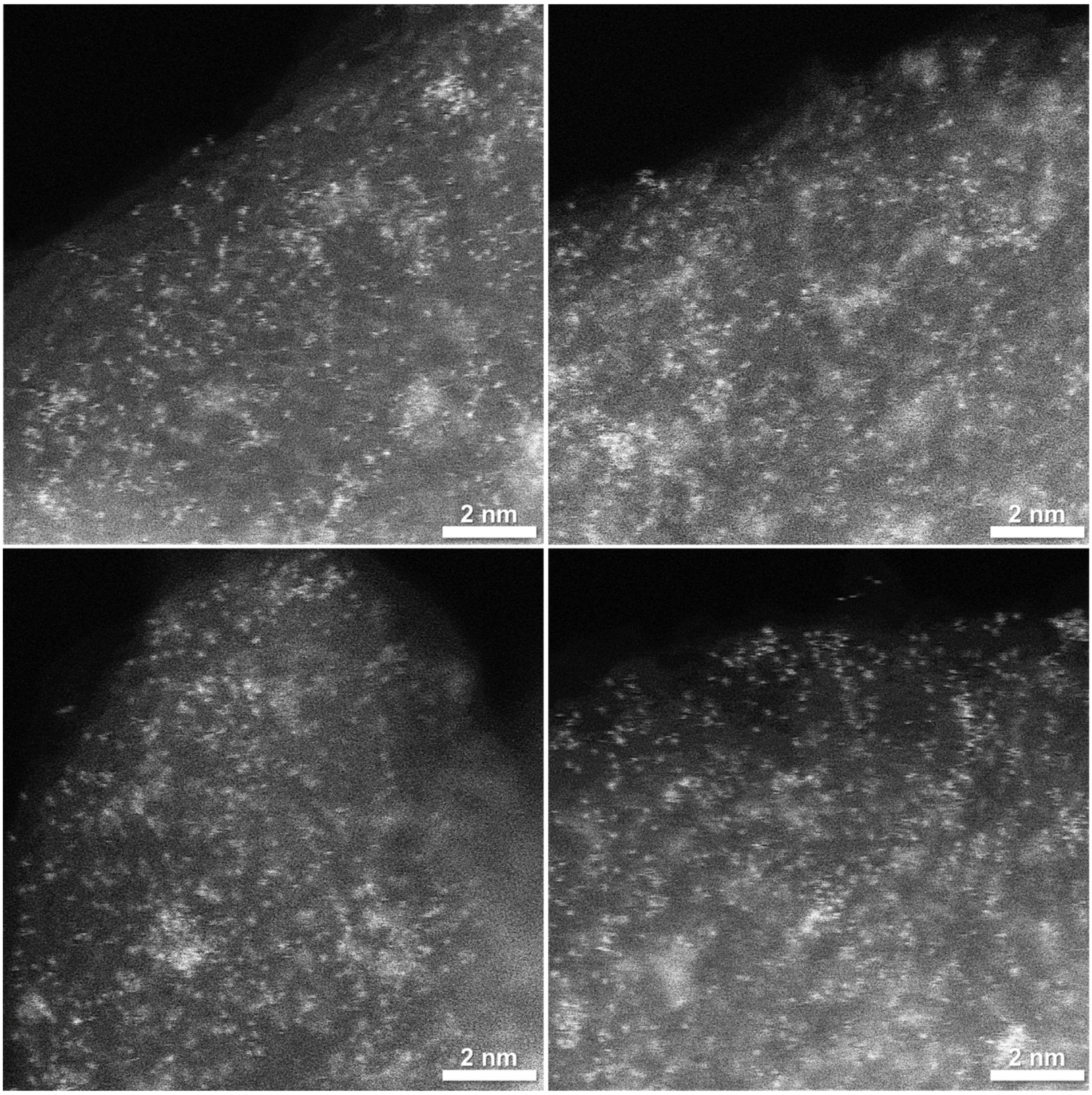

Supplementary Figure 2 | HAADF-STEM images of Pt/HSC. The images taken at different domains indicated mainly the presence of atomically dispersed Pt species on HSC support. In general, atomically dispersed metal species are unstable under the irradiation of an electron beam[1,2]. For instance, Kim *et al.* reported that TEM-invisible Pt species supported on sulfur-functionalized carbon nanotubes rapidly agglomerated to visible Pt clusters under high energy electron beam irradiation (300 kV)[3]. Under the similar conditions, the atomically dispersed Pt in Pt/HSC showed thermal vibrations and hopping to nearby sites, but notably, they were highly stable against metal sintering (see Supplementary Movie online). Therefore, it can be concluded that the S-moieties on the carbon support surface efficiently stabilize the atomically dispersed Pt species.

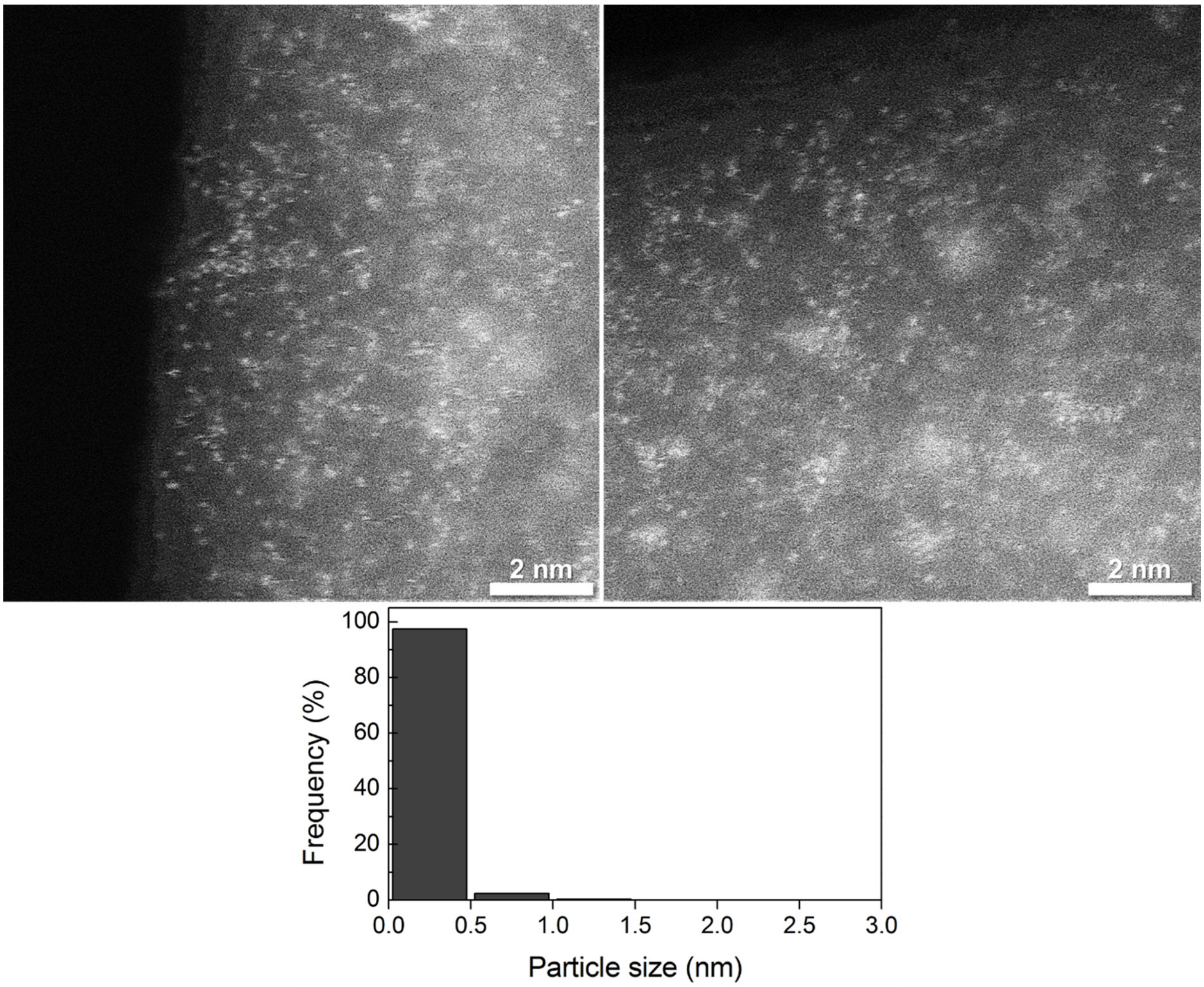

Supplementary Figure 3 | HAADF-STEM images of Pt/HSC kept under ambient air for 1 year. HAADF-STEM images of the Pt/HSC sample, which had been stored in ambient air for over a year (**top**). Histogram of the particle size distribution for Pt/HSC aged under ambient air for 1 year (**bottom**). The result indicates the predominant presence of atomically dispersed Pt species along with the minute formation of subnanometer Pt clusters, which were scarcely observed in the fresh Pt/HSC sample (Fig. 2i). However, the contrast between the fresh and aged samples is rather insignificant, indicating that the atomically dispersed Pt species in HSC are quite stable.

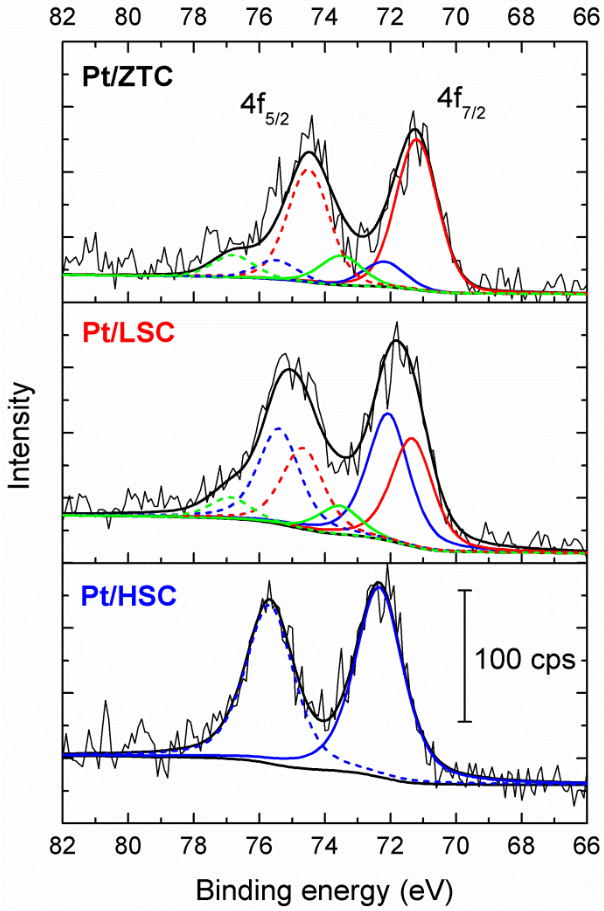

Supplementary Figure 4 | XPS-Pt_{4f} analysis of the Pt-supported carbon catalysts. The peaks were deconvoluted with Pt^0 (red lines), Pt^{2+} (blue lines), and Pt^{4+} (green lines) phases. The spin-orbit splitting and area ratio for $4f_{5/2}$ (dotted lines) and $4f_{7/2}$ (solid lines) peaks are 3.33 eV and 3:4, respectively.

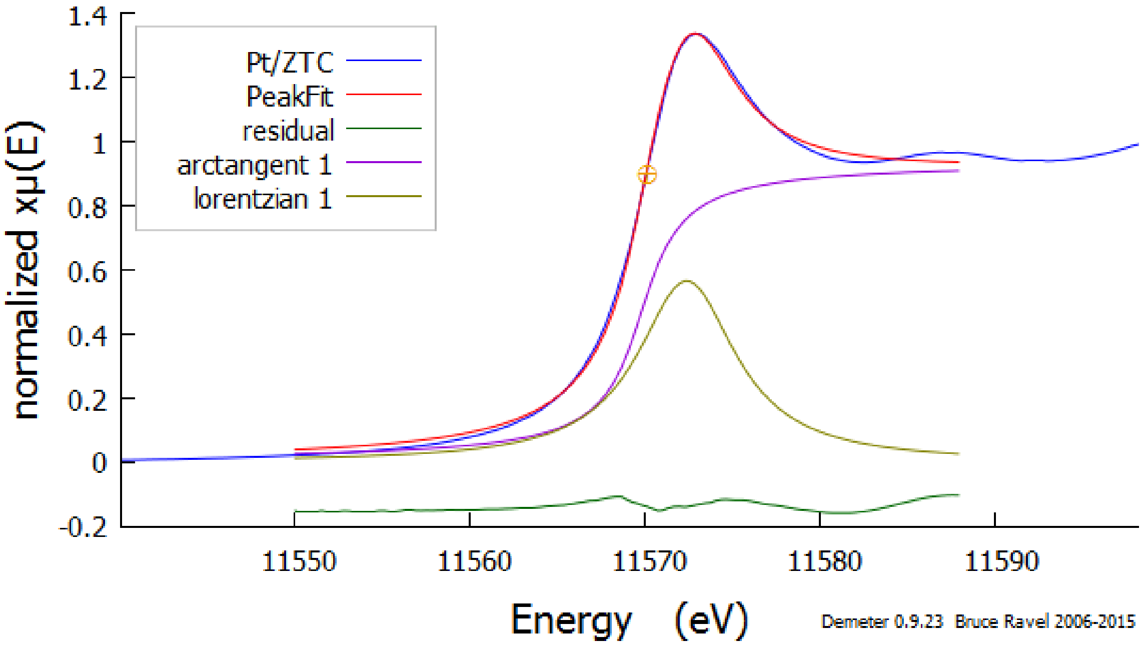

Supplementary Figure 5 | XANES fitting of Pt/ZTC. The analysis was carried out using arctangent and Lorentzian functions to fit the Fermi level and white line position.

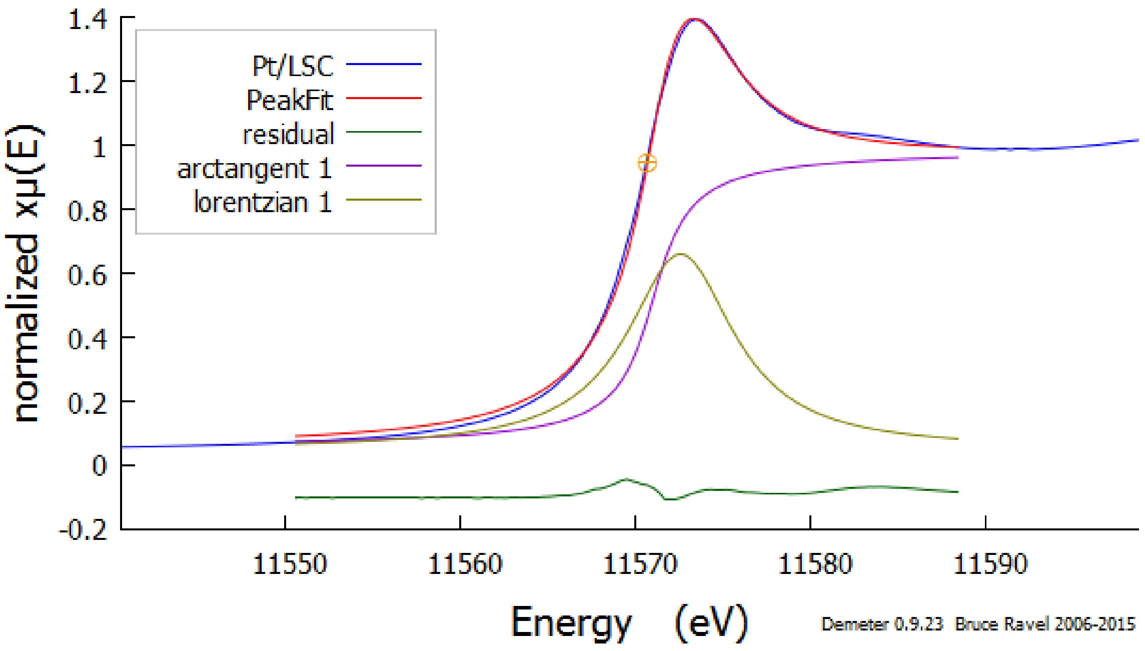

Supplementary Figure 6 | XANES fitting of Pt/LSC. The analysis was carried out using arctangent and Lorentzian functions to fit the Fermi level and white line position.

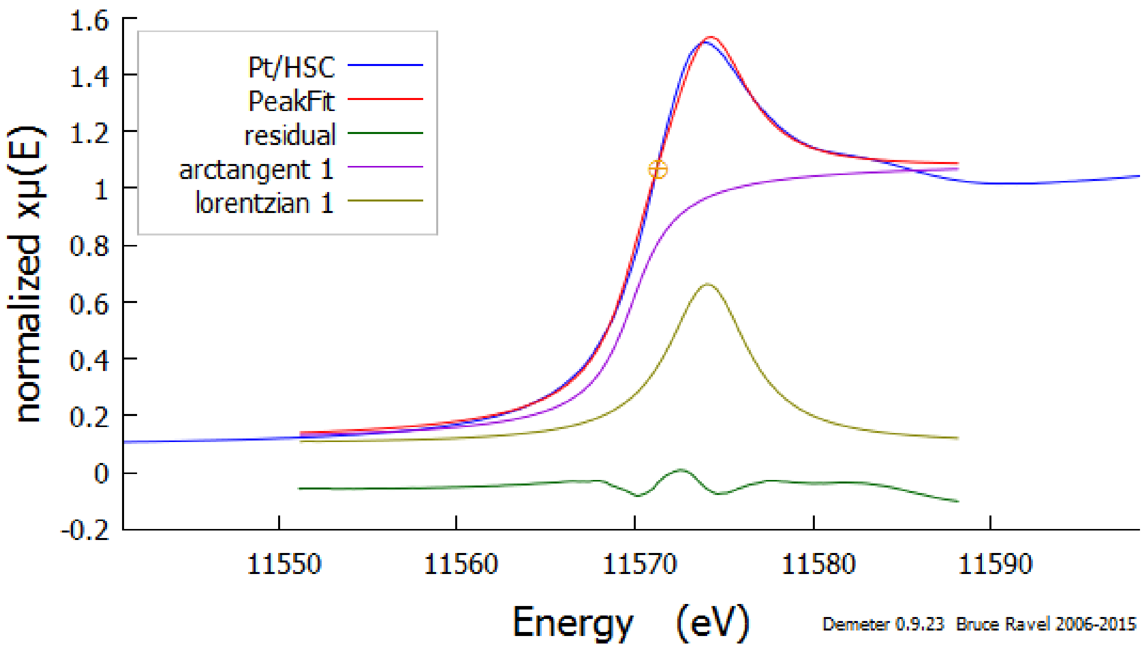

Supplementary Figure 7 | XANES fitting of Pt/HSC. The analysis was carried out using arctangent and Lorentzian functions to fit the Fermi level and white line position.

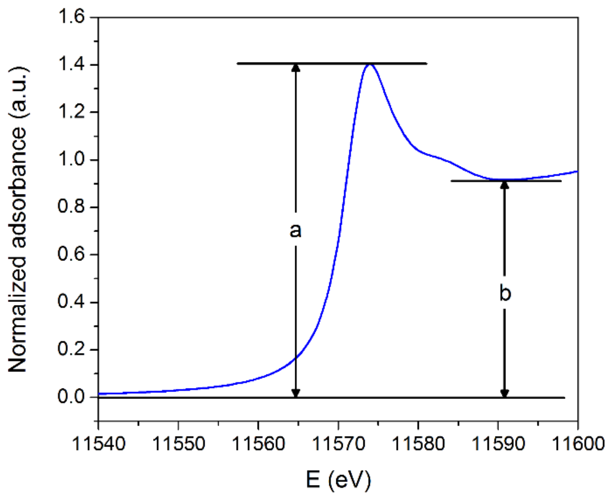

Supplementary Figure 8 | XANES of Pt/HSC. The white line height ratio (a/b) was found to be 1.52, which exactly corresponds to that of Pt^{2+} (1.52 ± 0.08) reported by M. D. Hall *et al.*[4].

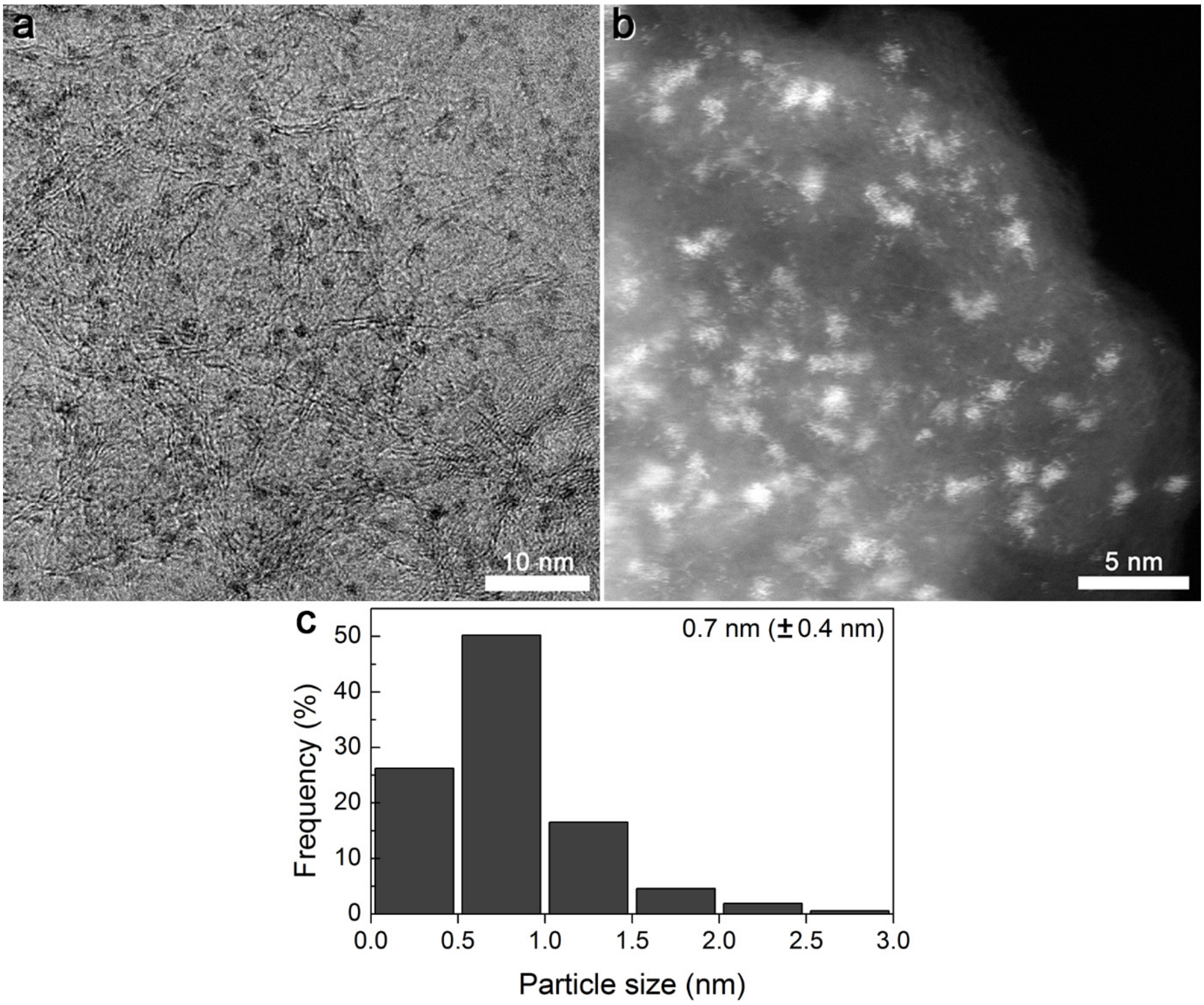

Supplementary Figure 9 | Structures of the Pt species on mesoporous S-doped carbon. a, TEM images indicating the presence of sub-nanometer Pt clusters in mesoporous S-doped carbon synthesized using SBA-15 as a sacrificial solid template. **b-c**, Atomic resolution HAADF-STEM image (**b**) and histogram of the particle size distribution (**c**). The results indicate that the mesoporous S-doped carbon mainly contains Pt clusters in addition to some atomically dispersed Pt species.

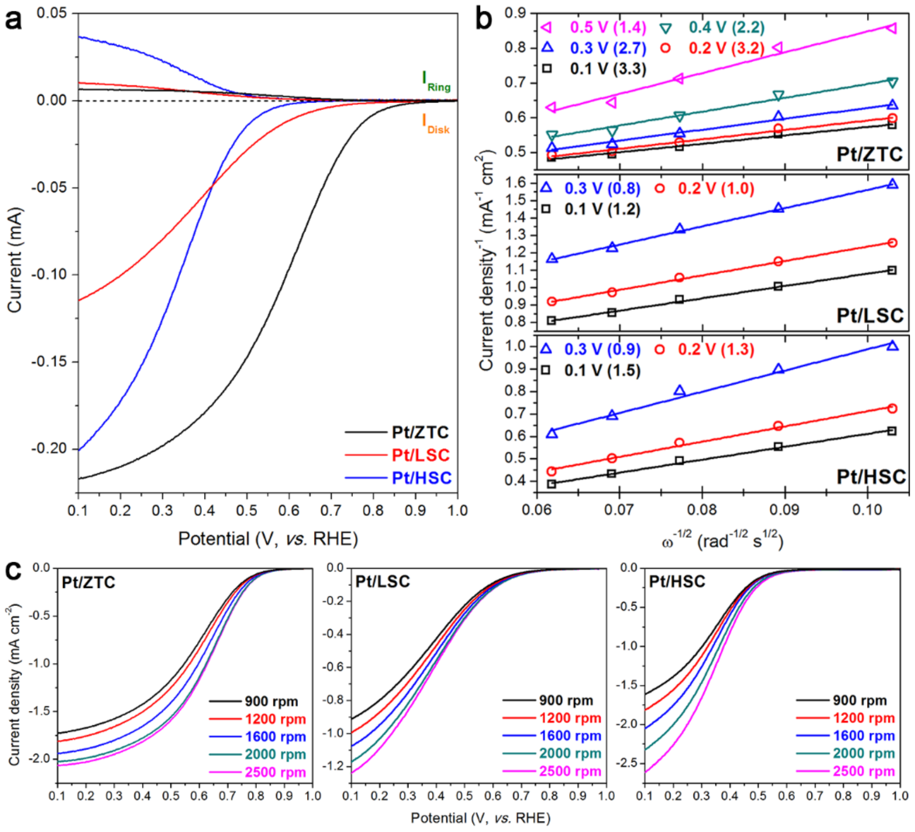

Supplementary Figure 10 | RRDE analysis of the Pt-supported carbon catalysts. a, Currents measured at ring (Pt ring potential: 1.2 V_{RHE}) and disk electrodes in ORR with 900 rpm rotation speed. **b**, Koutecky-Levich (K-L) plots obtained from Pt/ZTC, Pt/LSC, and Pt/HSC catalysts. Calculated n values at each potential were noted in parenthesis in the figures. **c**, ORR polarization curves of Pt/ZTC, Pt/LSC, and Pt/HSC at various rotation speeds. All the electrochemical measurements were performed in a 0.1 M $HClO_4$ electrolyte.

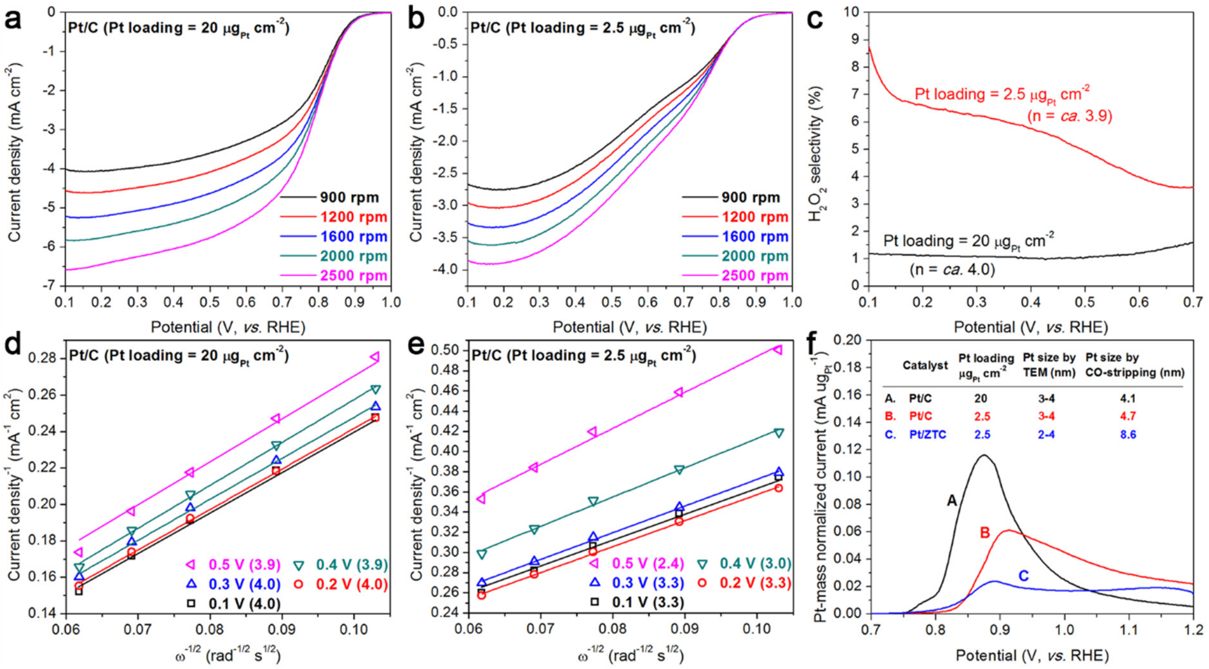

Supplementary Figure 11 | Effects of Pt loading on the K-L plots. a-b, ORR polarization curves of commercial Pt/C catalysts (Johnson Matthey, 20 wt% Pt) with different Pt loadings of 20 (**a**) and 2.5 μg_{Pt} cm^{-2} (**b**). **c**, H_2O_2 production selectivity of the commercial Pt/C catalysts, estimated by RRDE experiments (Pt ring potential: 1.2 V_{RHE}). **d-e**, K-L plots obtained from the commercial Pt/C catalysts with different Pt loadings of 20 (**d**) and 2.5 μg_{Pt} cm^{-2} (**e**). Calculated n values at each potential were noted in parenthesis in the figures. **f**, CO-stripping voltammetry results of the commercial Pt/C catalysts (Pt loadings = 20 and 2.5 μg_{Pt} cm^{-2}) and Pt/ZTC (Pt loadings = 2.5 μg_{Pt} cm^{-2}). The currents from CO oxidation were normalized by Pt-mass for comprehensive comparison among the data. Pt particle sizes calculated from TEM and CO-stripping voltammetry were summarized as an inset table. All the electrochemical measurements were performed in a 0.1 M $HClO_4$ electrolyte.

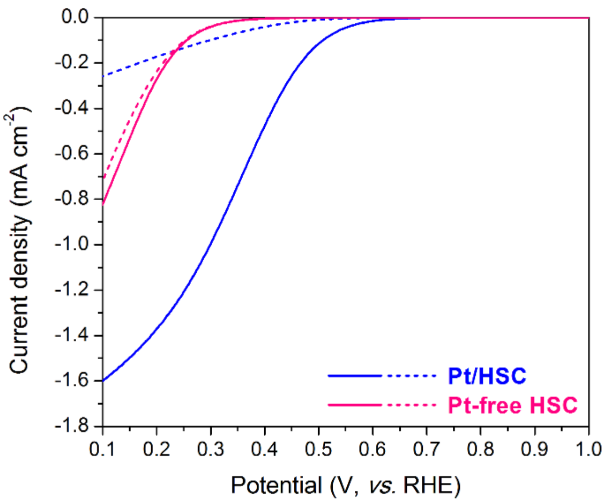

Supplementary Figure 12 | ORR activity of Pt-free HSC and that of Pt/HSC under CN^{-}-poisoning. All the experiments were examined in an O_2-saturated 0.1 M $HClO_4$ electrolyte with 900 rpm rotation speed. In the CN^{-}-poisoning experiment (dotted line), 10 mM KCN was additionally dissolved in the electrolyte. For comparison, ORR activity in a pure 0.1 M $HClO_4$ electrolyte without KCN is also described (solid line).

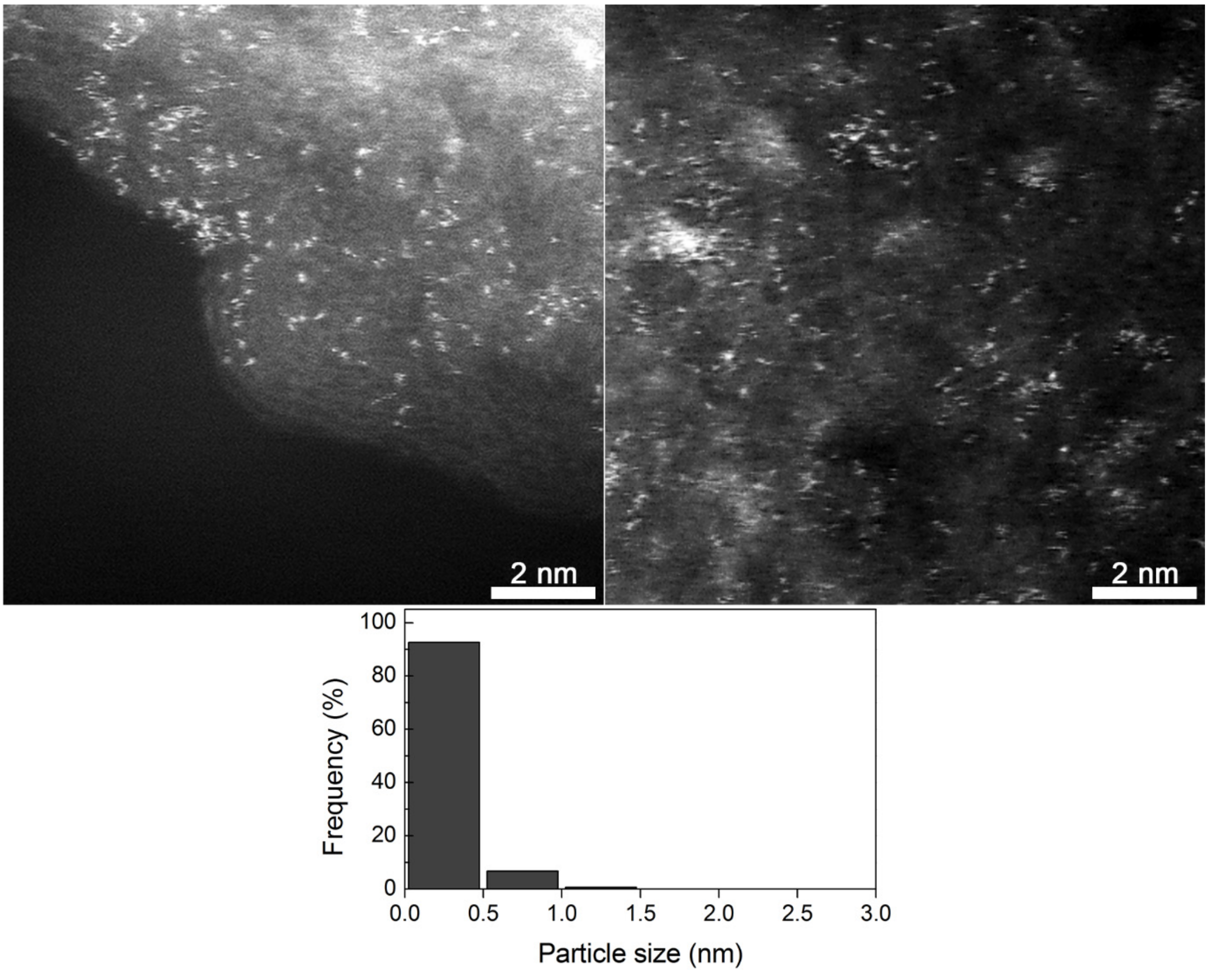

Supplementary Figure 13 | A HAADF-STEM image of the Pt/HSC after repeated cell operations. After 5 cycles of 2 h operations in the H-cell (Fig. 3f), Pt/HSC was collected from a cathode electrode and then HAADF-STEM image was taken at 300 kV accelerating voltage (**top**). Histogram of the particle size distribution for Pt/HSC after repeated cell operations (**bottom**). The result indicates the minute formation of subnanometer Pt clusters, while most of the atomically dispersed Pt species were preserved after the operations.

a. Model structure #1

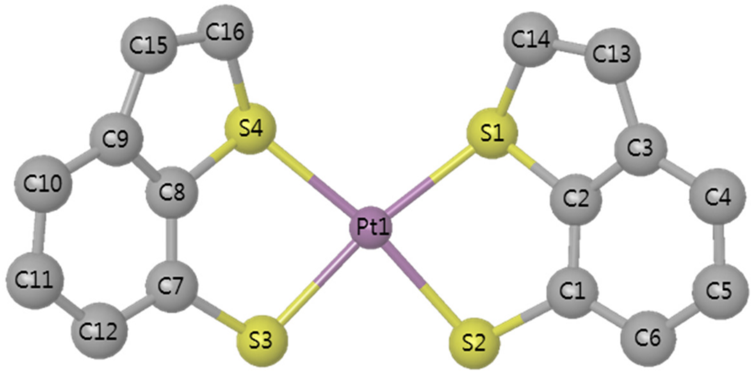

b. Model structure #2

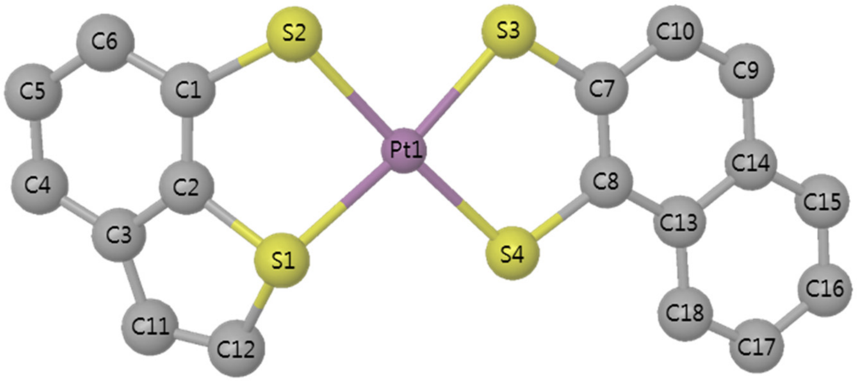

c. Model structure #3

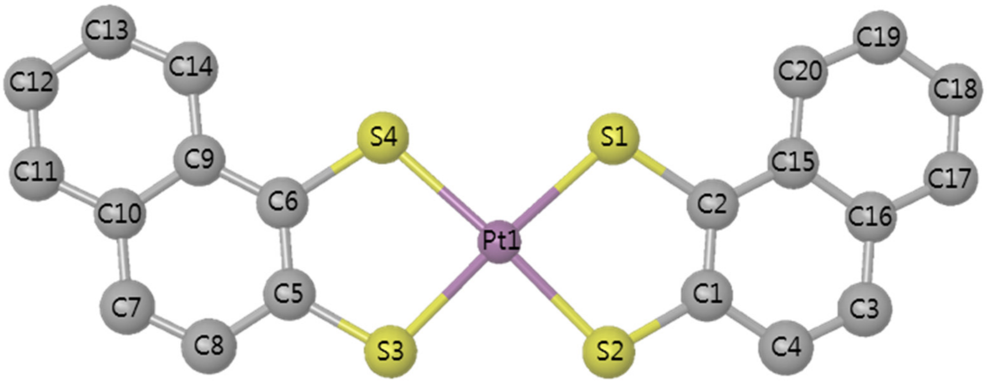

Supplementary Figure 14 | Pt-S_4 model structures. Three possible model structures of Pt-S_4 including **a**, [Structure #1] Pt ligated by two thiophenes (S1/S4) and two thiolates (S2/S3), **b**, [Structure #2] Pt ligated by one thiophene (S1), one thiol (S4), and two thiolates (S2/S3), and **c**, [Structure #3] Pt ligated by two thiols (S1/S4) and two thiolates (S2/S3). Energy of the structures was minimized by using DFT calculations.

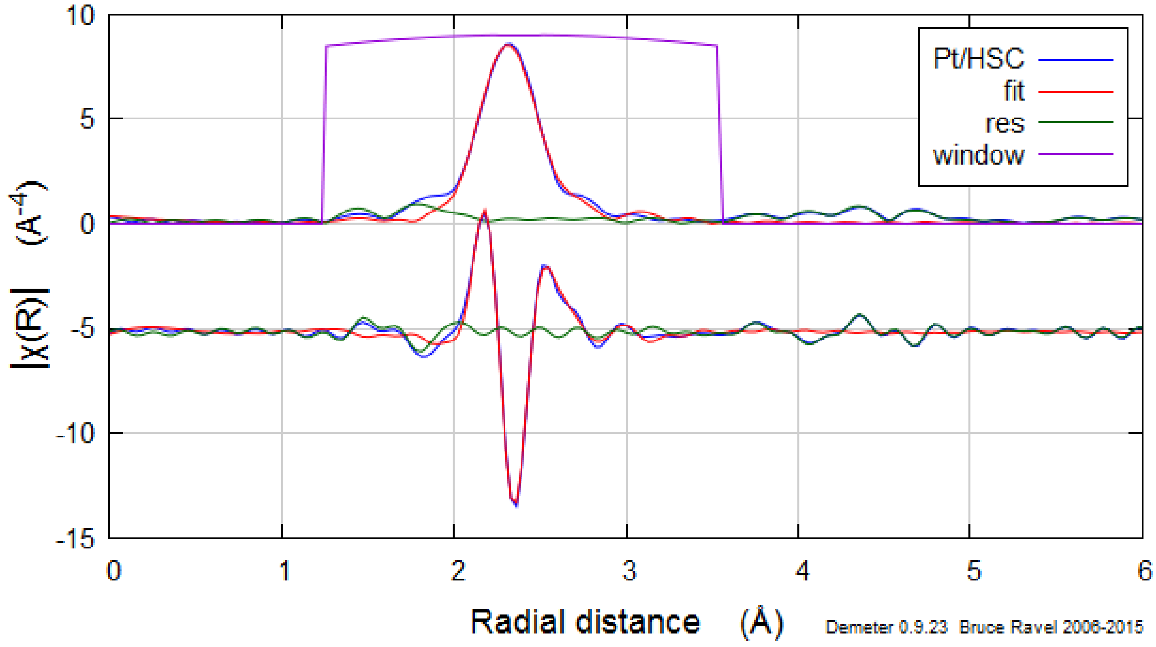

Pair	CN	R (Å)	$\Delta\sigma^2$ (Å^2)	ΔE_0 (eV)	R-factor
**Pt-S**	3.9 (± 0.3)	2.29 (± 0.01)	0.0060 (± 0.0007)		
**Pt-C2/8**	0.6 (± 0.7)	2.96 (± 0.04)	0.0005 (± 0.0043)	5.5 (± 1.0)	0.0053
**Pt-C1/7**	3.0 (± 0.1)	3.32 (± 0.15)	0.0447 (± 0.0501)		

Supplementary Figure 15 | EXAFS fitting results of Pt-S_4 model structure #1. Multi-shell fitting results (Δk = 2 – 16 Å^{-1}) of the experimental EXAFS spectrum using suggested model structure #1, *i.e.* Pt ligated by two thiophenes and two thiolates.

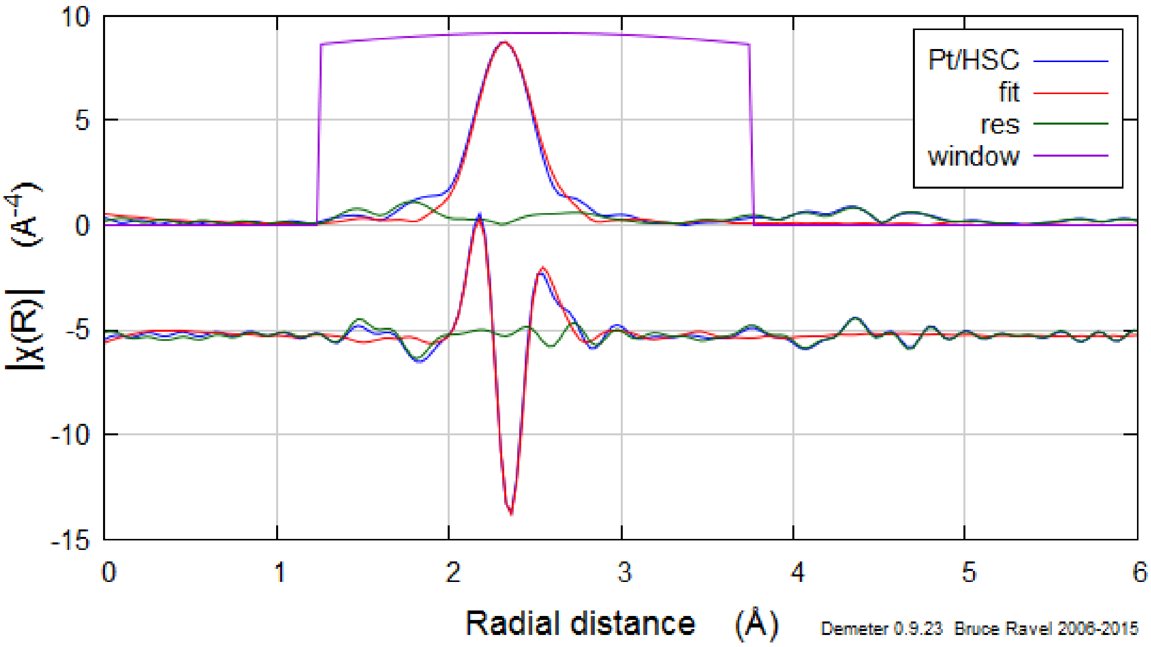

Pair	CN	R (Å)	Δσ^2 (Å^2)	ΔE$_0$ (eV)	R-factor
**Pt-S**	4.1 (± 0.5)	2.30 (± 0.01)	0.0066 (± 0.0011)	7.6 (± 1.2)	0.0122
**Pt-C2**	3.0 (± 0.1)	2.94 (± 0.07)	0.0119 (± 0.0154)		
**Pt-C1/7**	2.8 (± 0.1)	3.17 (± 0.12)	0.0139 (± 0.0371)		
**Pt-C8**	3.0 (± 0.1)	3.40 (± 0.13)	0.0172 (± 0.0371)		

Supplementary Figure 16 | EXAFS fitting results of Pt-S$_4$ model structure #2. Multi-shell fitting results (Δk = 2 – 16 Å^{-1}) of the experimental EXAFS spectrum using suggested model structure #2, *i.e.* Pt ligated by one thiophene, one thiol, and two thiolates.

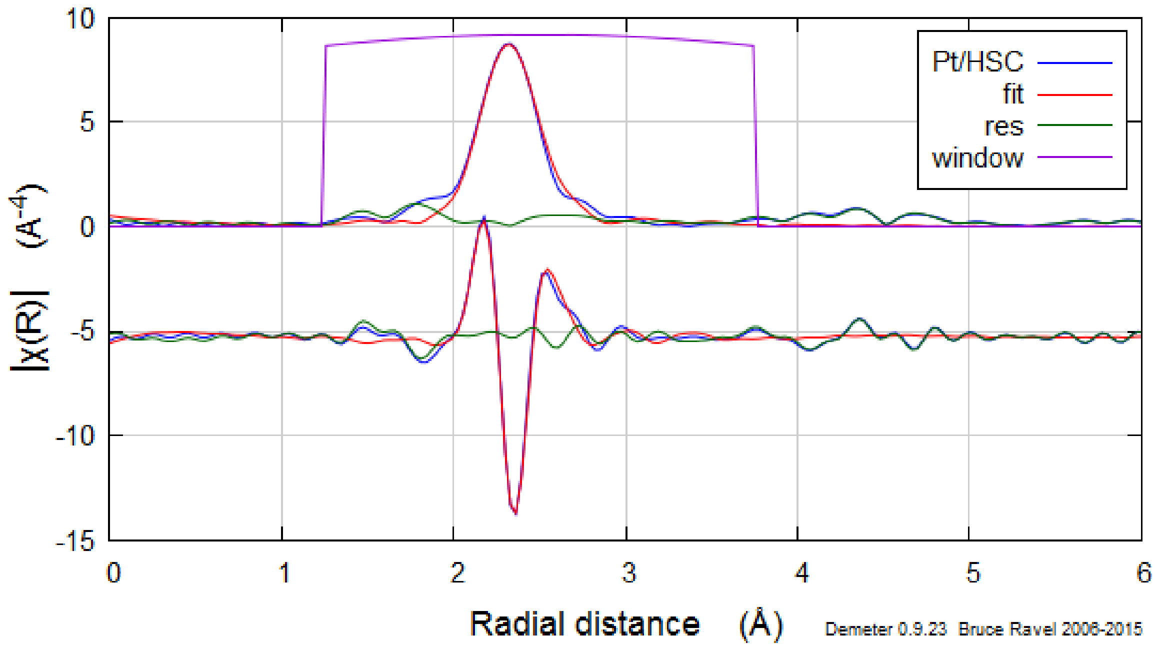

Pair	CN	R (Å)	$\Delta\sigma^2$ (Å^2)	ΔE_0 (eV)	R-factor
**Pt-S**	4.1 (± 0.4)	2.30 (± 0.01)	0.0067 (± 0.0010)	7.2 (± 1.0)	0.0138
**Pt-C1/5**	3.0 (± 0.1)	2.99 (± 0.04)	0.0129 (± 0.0084)		
**Pt-C2/6**	3.0 (± 0.1)	3.30 (± 0.06)	0.0149 (± 0.0123)		

Supplementary Figure 17 | EXAFS fitting results of Pt-S_4 model structure #3. Multi-shell fitting results (Δk = 2 – 16 Å^{-1}) of the experimental EXAFS spectrum using suggested model structure #3, *i.e.* Pt ligated by two thiols and two thiolates.

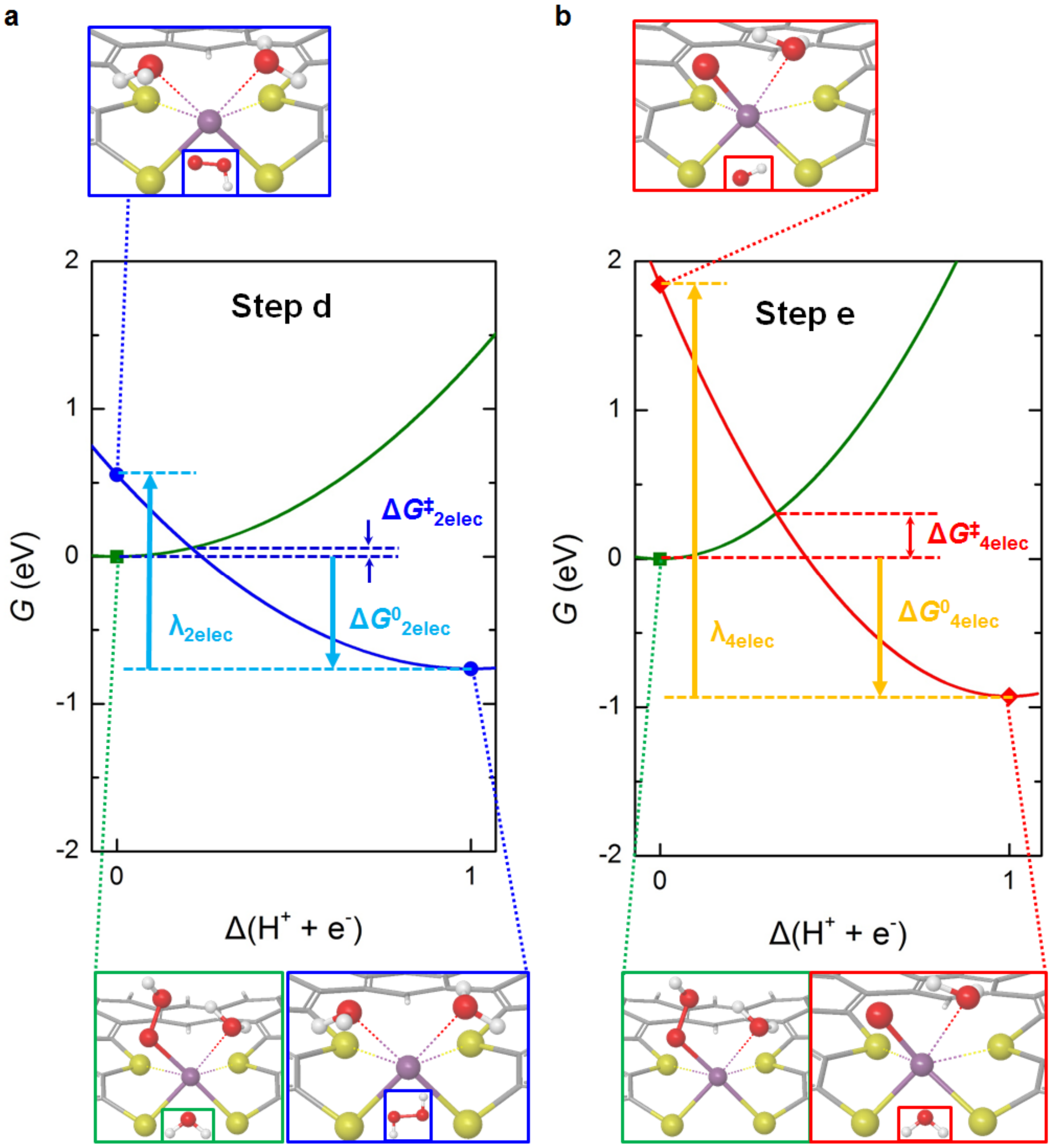

Supplementary Figure 18 | Marcus parabolas for the 2nd proton-coupled electron transfer (PCET) steps. Under the quadratic assumption, Marcus curves were constructed by the calculation of the Gibbs free energy of reaction, ΔG^0, the Gibbs free energy of activation, $\Delta G^{\ddagger}$, and the reorganization energy of a product structure by the proton-coupled electron transfer (referring to the reaction steps in Fig. 4). The reaction coordinate is chosen as the number of transferred pairs of proton and electron. At zero bias potential, the PCET to the OOH occurs with three times less the cost of barrier energy in **a**, the 2-electron pathway than in **b,** the 4-electron pathway. The atomistic structures employed for computing relative energetics of ΔG^0 (the Gibbs free energy of reaction with no bias potential) and λ_o (the reorganization energy) are shown in insets, where molecule in the subinset means that it is in aqueous state separated from the catalytic active center.

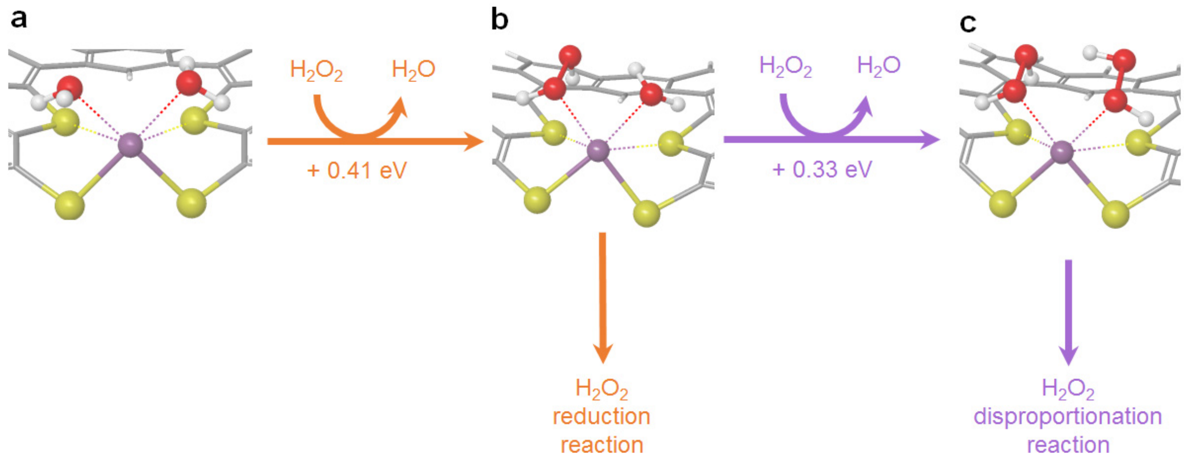

Supplementary Figure 19 | Decomposition pathways of H_2O_2. a, Pt catalytic active center can decompose H_2O_2 molecules only after **b**, one H_2O_2 molecule is adsorbed to Pt for peroxide reduction reaction (PRR) and **c**, two H_2O_2 molecules are adsorbed to Pt for peroxide disproportionation reaction (PDR), both of which requires a substantial thermodynamic costs of 0.41 eV and 0.33 eV, respectively.

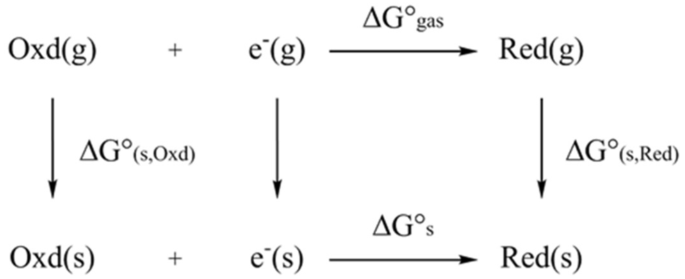

Supplementary Figure 20 | Born-Haber cycle. We used thermodynamic cycles to compute the Gibbs free energy for the reaction of solvated species.

Supplementary Tables

Supplementary Table 1 | Surface areas and pore volumes of the ZTC, LSC, and HSC.

Sample	S_{BET}[a] (m^2 g^{-1})	V_{micro}[b] (mL g^{-1})	V_{meso}[c] (mL g^{-1})	V_{total}[d] (mL g^{-1})
**ZTC**	2630	1.04	0.26	1.30
**LSC**	2420	0.99	0.16	1.15
**HSC**	2770	0.95	0.37	1.32

[a] BET surface areas (S_{BET}) were determined in the P/P_0 range of 0.05 – 0.15. [b] Micropore volumes (V_{micro}) were determined by the Dubinin–Astakhov method. [c] Mesopore volumes (V_{meso}) were calculated by 'V_{total}-V_{micro}'. [d] Total pore volumes (V_{total}) were evaluated at $P/P_0 = 0.95$.

Supplementary Table 2 | EXAFS fitting results of the prepared catalysts[a].

Sample	Shell	CN	R (Å)	$\Delta\sigma^2$ ($Å^2$)	ΔE_0 (eV)	R-factor[b]
**Pt/ZTC**	Pt-Pt	9.2 (± 0.7)	2.75 (± 0.01)	0.0068 (± 0.0003)	8.4 (± 0.9)	0.0120
**Pt/LSC**	Pt-Pt	3.3 (± 1.1)	2.71 (± 0.01)	0.0103 (± 0.0020)	7.4 (± 1.6)	0.0097
	Pt-S	2.4 (± 0.3)	2.30 (± 0.01)	0.0060 (± 0.0010)		
**Pt/HSC**	Pt-S	3.8 (± 0.6)	2.29 (± 0.01)	0.0067 (± 0.0013)	5.9 (± 1.8)	0.0279
**Pt/HSC[c]**	Pt-Pt	−0.4 (± 0.9)	2.79 (± 0.06)	0.0097 (± 0.0020)	5.7 (± 0.9)	0.0050
	Pt-S	3.4 (± 0.3)	2.29 (± 0.01)	0.0050 (± 0.0007)		

[a] EXAFS fitting was carried out up to the second shell (Δk = 3 – 14 $Å^{-1}$ for Pt/ZTC and Pt/LSC, and Δk = 3 – 12 $Å^{-1}$ for Pt/HSC). [b] The R-factor was estimated during the refinement as implanted in IFEFFIT program. [c] EXAFS fitting results considering both Pt-Pt and Pt-S coordinations. The double-shell fitting gave a very small and negative coordination number for Pt-Pt (CN = −0.4 ± 0.9), which is not physically much meaningful. This result confirms the insignificant presence of Pt clusters in Pt/HSC sample.

Supplementary Table 3 | XANES analysis results of the prepared catalysts[a].

Sample	Continuum level E_0 (eV)	White line position E_0 (eV)	White line Area (a.u.)	R-factor
**Pt/ZTC**	11568.86 (± 0.23)	11572.80 (± 0.17)	3.93	0.0009
**Pt/LSC**	11569.61 (± 0.15)	11573.37 (± 0.10)	4.29	0.0004
**Pt/HSC**	11569.86 (± 0.25)	11574.09 (± 0.13)	4.83	0.0007

[a] XANES was analyzed using the peak fitting routine implemented in Athena.

Supplementary Note

Supplementary Note 1: To measure H_2O_2 production selectivity in ORR, RRDE experiments (Supplementary Fig. 10a) and Koutecky-Levich (K-L) plots (Supplementary Fig. 10b) were obtained in an O_2-saturated 0.1 M $HClO_4$ electrolyte.

$$\frac{1}{J}=\frac{1}{J_{\mathrm{k}}}+\frac{1}{J_{\mathrm{d}}}=\frac{1}{J_{\mathrm{k}}}+\frac{1}{nK\omega^{1/2}} \quad \text{(Supplementary equation 1)}$$

$$K=0.62nFD^{2/3}\upsilon^{-1/6}C \quad \text{(Supplementary equation 2)}$$

where J is the total current density, J_{k} is the kinetic current density, J_{d} is the diffusion current density, n is the number of electron transferred, ω is the rotation speed, F is the Faraday constant (96485 C mol^{-1}), D is the diffusion coefficient of O_2 (1.93×10^{-5} cm^2 s^{-1} in 0.1 M $HClO_4$), v is the kinematic viscosity of solution (1.009×10^{-2} cm^2 s^{-1}), and C is the saturated concentration of O_2 (1.26×10^{-6} mol cm^{-3})[5].

ORR polarization curves do not reach the theoretical diffusion-limited current densities within an operating potential window at various rotating speeds (*e.g.*, 4.2 and 2.1 mA cm^{-2} for 4- and 2-electron reductions at a 900 rpm rotating speed, respectively) (Supplementary Fig. 10c), and more obviously, n values calculated from slopes of the K-L plots are significantly underestimated (Supplementary Fig. 10b) compared to those from RRDE measurements and other measurements on standard Pt catalysts (see also Supplementary Fig. 11). Furthermore, the K-L slope shows a strong potential dependence, so that the n value increases as potential decreases. This untypical electrochemical result can be ascribed to the special behavior of these types of catalyst, in particular to the highly microporous and high surface area support with a low Pt loading. The Pt loading on the electrode is very low as 2.5 μg_{Pt} cm^{-2} (= 50 $\mu g_{catalyst}$ cm^{-2} × 5 wt% Pt), which could introduce artefacts in the applied classical evaluation of RDE data[6]. Moreover, this is even more aggravated by an inaccessibility of active Pt sites within the microporous support structure, which is likely to occur on these types of materials. A large portion of Pt nanoparticles and active single-sites are located inside the micropores of zeolite-templated carbons. CO-stripping voltammetry results support the low utilization of Pt nanoparticles for instance for the Pt/ZTC, for which the electroactive surface area (EAS) is significantly lower than that of commercial Pt/C (Supplementary Fig. 11f); Pt particle size calculated from the CO-stripping result is 8.6 nm, which is much larger than the actual particle size of *ca.* 4 nm as confirmed by TEM results (Fig. 2a). Because of the low kinetic activity resulting from the low Pt loading and the highly microporous carbon structure, the K-L plot seems to be less suitable for calculating H_2O_2 selectivity of the prepared catalysts. In this study, therefore, the H_2O_2 selectivity was calculated by RRDE experiments. Overall it seems that the fully quantitative thin-film RDE evaluation is not feasible anymore when the active material is in such a dispersed state. Nevertheless, the semi-quantitative information about onset potentials as well as H_2O_2 production detected at the ring still holds, and provides an excellent first insight into the unique behavior of the materials.

Note that in order to confirm that the electrochemical behaviors observed for the zeolite-templated carbons is only due to the dispersed state of the Pt active sites and not an artefact of impurities, we performed additional

reference experiments using a commercial Pt/C catalyst (Johnson matthey, 20 wt% Pt) with two different Pt loadings of 20 and 2.5 μg_{Pt} cm^{-2} (Supplementary Fig. 11). The ORR on the Pt/C electrode with a high Pt loading of 20 μg_{Pt} cm^{-2} reaches the theoretical values of J_d, as expected, and RRDE and K-L results show almost similar value of *ca.* n = 4. Similar to the results from Pt/ZTC, however, the Pt/C electrode with a low Pt loading of 2.5 μg_{Pt} cm^{-2} does not reach the theoretical values of J_d, and n values calculated from K-L slope (n = 2.4 − 3.3) are also underestimated compared to that from RRDE (*ca.* n = 3.9) with a strong potential-dependent behavior. Moreover, it was examined if this deviation from ideal behavior is due to an effect of organic impurities in the electrolyte, which could be enhanced considering the low amount of active sites[7]. However, potential cycling to more positive potentials did not result in any activation of the zeolite-templated carbon materials, as would be expected in such a case and has been shown for other Pt-based materials. This confirms the previous assumption that despite the break-down of the thin-film RDE evaluation capabilities, semi-quantitative data on H_2O_2 production can still be extracted and conclusions about the potential of the unique catalysts are valid. This is validated in the manuscript (see also Fig. 3) by additional and complementary investigations about PRR, PDR and extended H-cell tests.

Supplementary References

1 Uzun, A., Ortalan, V., Hao, Y. L., Browning, N. D. & Gates, B. C. Nanoclusters of gold on a high-area support: Almost uniform nanoclusters imaged by scanning transmission electron microscopy. *ACS Nano* **3,** 3691-3695 (2009).

2 Uzun, A., Ortalan, V., Browning, N. D. & Gates, B. C. A site-isolated mononuclear iridium complex catalyst supported on MgO: Characterization by spectroscopy and aberration-corrected scanning transmission electron microscopy. *J. Catal.* **269,** 318-328 (2010).

3 Kim, Y.-T. *et al.* Fine size control of platinum on carbon nanotubes: From single atoms to clusters. *Angew. Chem. Int. Ed.* **45,** 407-411 (2006).

4 Hall, M. D., Foran, G. J., Zhang, M., Beale, P. J. & Hambley, T. W. XANES determination of the platinum oxidation state distribution in cancer cells treated with platinum(IV) anticancer agents. *J. Am. Chem. Soc.* **125,** 7524-7525 (2003).

5 Tiwari, J. N. *et al.* Stable platinum nanoclusters on genomic DNA-graphene oxide with a high oxygen reduction reaction activity. *Nat. Commun.* **4,** 2221 (2013).

6 Mayrhofer, K. J. J. *et al.* Measurement of oxygen reduction activities via the rotating disc electrode method: From Pt model surfaces to carbon-supported high surface area catalysts. *Electrochim. Acta* **53,** 3181-3188 (2008).

7 Katsounaros, I., Meier, J. C. & Mayrhofer, K. J. J. The impact of chloride ions and the catalyst loading on the reduction of H_2O_2 on high-surface-area platinum catalysts. *Electrochim. Acta* **110,** 790-795 (2013).
